# Real-Time Imaging Reveals the Dynamics of Leukocyte Behaviour during Experimental Cerebral Malaria Pathogenesis

**DOI:** 10.1371/journal.ppat.1004236

**Published:** 2014-07-17

**Authors:** Saparna Pai, Jim Qin, Lois Cavanagh, Andrew Mitchell, Fatima El-Assaad, Rohit Jain, Valery Combes, Nicholas H. Hunt, Georges E. R. Grau, Wolfgang Weninger

**Affiliations:** 1 Immune Imaging Laboratory, The Centenary Institute, Newtown, Sydney, New South Wales, Australia; 2 Sydney Medical School, University of Sydney, Sydney, New South Wales, Australia; 3 Vascular Immunology Unit, Discipline of Pathology, Sydney Medical School, University of Sydney, Camperdown, Sydney, New South Wales, Australia; 4 Molecular Immunopathology Unit, Discipline of Pathology, Sydney Medical School and Bosch Institute, University of Sydney, Camperdown, Sydney, New South Wales, Australia; 5 Discipline of Dermatology, University of Sydney, Sydney, New South Wales, Australia; 6 Department of Dermatology, Royal Prince Alfred Hospital, Camperdown, Sydney, New South Wales, Australia; Faculdade de Medicina da Universidade de Lisboa, Portugal

## Abstract

During experimental cerebral malaria (ECM) mice develop a lethal neuropathological syndrome associated with microcirculatory dysfunction and intravascular leukocyte sequestration. The precise spatio-temporal context in which the intravascular immune response unfolds is incompletely understood. We developed a 2-photon intravital microscopy (2P-IVM)-based brain-imaging model to monitor the real-time behaviour of leukocytes directly within the brain vasculature during ECM. Ly6C^hi^ monocytes, but not neutrophils, started to accumulate in the blood vessels of *Plasmodium berghei* ANKA (PbA)-infected MacGreen mice, in which myeloid cells express GFP, one to two days prior to the onset of the neurological signs (NS). A decrease in the rolling speed of monocytes, a measure of endothelial cell activation, was associated with progressive worsening of clinical symptoms. Adoptive transfer experiments with defined immune cell subsets in recombinase activating gene (RAG)-1-deficient mice showed that these changes were mediated by *Plasmodium*-specific CD8^+^ T lymphocytes. A critical number of CD8^+^ T effectors was required to induce disease and monocyte adherence to the vasculature. Depletion of monocytes at the onset of disease symptoms resulted in decreased lymphocyte accumulation, suggesting reciprocal effects of monocytes and T cells on their recruitment within the brain. Together, our studies define the real-time kinetics of leukocyte behaviour in the central nervous system during ECM, and reveal a significant role for *Plasmodium*-specific CD8^+^ T lymphocytes in regulating vascular pathology in this disease.

## Author Summary

Cerebral malaria (CM) is a severe complication of *Plasmodium falciparum* infection that takes a significant toll on human life. Blockage of the brain blood vessels contributes to the clinical signs of CM, however we know little about the precise pathological events that lead to this disease. To this end, studies in *Plasmodium*-infected mice, that also develop a similar fatal disease, have proven useful. These studies have revealed an important role for leukocytes not so much in protecting but rather promoting pathology in the brain. To better understand leukocyte behaviour during experimental CM, we established a brain-imaging model that allows us to ‘peek’ into the brain of living mice and watch immunological events as they unfold. We found that worsening of disease was accompanied by an accumulation of monocytes in the blood vessels. Monocyte accumulation was regulated by activated CD8^+^ T cells but only when present in critical numbers. Monocyte depletion resulted in reduced T cell trafficking to the brain, but this did not result in improved disease outcome. Our studies reveal the orchestration of leukocyte accumulation in real time during CM, and demonstrate that CD8^+^ T cells play a crucial role in promoting clinical signs in this disease.

## Introduction

CM is a severe complication of *Plasmodium falciparum* infection and is responsible for an estimated 627,000 deaths annually, particularly among children [1]. CM is strongly associated with parasitised red blood cell (pRBC) sequestration in the vasculature of the brain [2], [3]. The resultant obstruction of blood flow has been proposed as a potential pathological mechanism leading to ischemia and hypoxia of the central nervous system (CNS) [4], [5], [6]. Although direct visualisation of the retinal vasculature in infected humans supports this notion [7], most neuropathological observations in humans are limited to post-mortem analysis of brain tissues. In addition, imaging approaches such as MRI do not resolve details of microcirculatory dysfunction or neuropathology in the intact brain [6], [8]. Therefore, we lack a dynamic view of the events leading to progressive CNS damage during CM.

The murine model of PbA-infection has proven useful in studying ECM pathogenesis [9], [10], [11], [12]. In the PbA model, leukocytes accumulate in the brain microcirculation when mice develop NS, similarly to human disease [12], [13], [14], [15]. However, to date no detailed real-time microscopic investigations have been conducted to assess the spatio-temporal dynamics of leukocyte behaviour in the brain during ECM development. Leukocyte imaging has been limited to visualising circulating cells using non-specific dyes or fluorescent antibodies [16], [17]. Thus, it is not known whether and how local inflammation within the brain microvasculature correlates with disease progression, and what factors control the sequestration of leukocytes in the blood vessels. In the present work, we used 2P-IVM [18] to quantitate and characterize specific leukocyte subsets *in situ* within the brain microvasculature in a dynamic manner.

Observations that athymic nude mice are protected from CM have implicated T cells in the pathogenesis of disease [19]. Indeed, several studies have documented that the CD8^+^ T cell subset does not protect but rather promotes NS during PbA infection. Mice depleted of or deficient in CD8^+^ T cells, β2-microglobulin, perforin or granzyme B are all protected from ECM establishing the role of effector T cell cytotoxicity in its pathogenesis [9], [13], [20], [21], [22], [23]. Further, antigen-specific cytotoxic T lymphocytes (CTL) sequester within the brain vasculature where they produce granzyme B to promote NS [20], [21], [24]. Nevertheless, the precise effects of CD8^+^ T cells on vascular neuropathology during CM are not entirely clear.

The primary goal of this study was to investigate the time-dependent development of vascular neuropathology during ECM *in situ* using 2P-IVM and to dissect the role of CD8^+^ T cells in regulating leukocyte trafficking within the CNS. Our study describes some of the precise events that lead up to vascular neuropathology during ECM development. We show that monocytes are a prominent cell type adhering to the microvasculature of the brain during ECM. These cells enhance recruitment of CD4^+^ and CD8^+^ T cells to the CNS vasculature but are not essential for disease. Further, we show that adhesive behaviour and rolling velocity of monocytes change depending on the disease stage and are regulated by the presence of *Plasmodium*-specific CD8^+^ T cells, which in contrast, are crucial for driving clinical disease.

## Results

### Ly6C^hi^ monocytes and CD8^+^ T lymphocytes sequester in the brain during ECM

**Figure 1:**
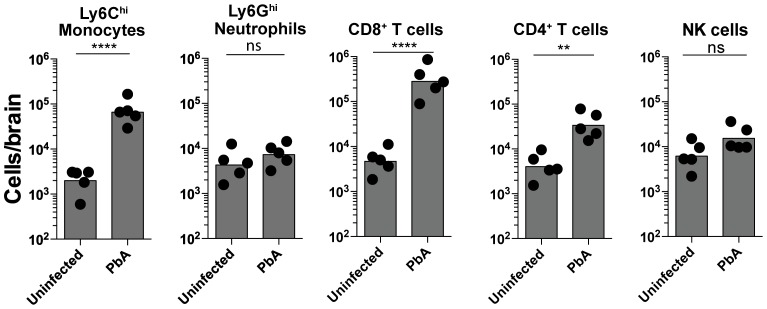
Monocytes and CD8^+^ T lymphocytes accumulate in the brain during ECM. Uninfected and PbA-infected C57BL/6 mice with NS were sacrificed on day 7 p.i. and brains harvested. Quantitative analysis of BSL is shown. Symbols represent individual animals. Bars represent the geometric mean. Data are a mean of 5 mice per group (n = 5). Monocytes [2309±489 versus 77,340±23090], Neutrophils [5432±1898 versus 8242±1928], CD8^+^ T lymphocytes [5520±1573 versus 365,960±134,268], CD4^+^ T lymphocytes [4792±1381 versus 40,240±11,872] and NK cells [7480±2235 versus 17,874±5262]. ns, not significant, **p<0.001, ****P<0.0001 (Unpaired t test).

To characterize and quantify brain sequestered leukocytes (BSL) during ECM, we performed flow cytometric analysis of brain single cell suspensions harvested from uninfected (UI) and PbA-infected C57BL/6 mice. PbA-infected mice included in this analysis had all developed NS (see Materials and Methods and Fig. S1 for disease scoring). CD11b^+^Ly6G^−^Ly6C^hi^ monocytes constituted 15.5±1.5% of total CD45^hi^ BSL in PbA-infected mice as compared to 4.3±0.6% in uninfected mice (Fig. S2A, C). CD8^+^ T cells constituted 51.9±2.6% and 10.9±1.7% and CD4^+^ T cells 6.4±0.7% and 9.3±1.5% of total CD45^hi^ BSL in PbA-infected and uninfected mice, respectively (Fig. S2B, C). This translated to a 33-fold increase in absolute monocyte numbers and a 66-fold and 8-fold increase in absolute CD8^+^ T and CD4^+^ T cell numbers, respectively, in PbA-infected mice (Fig. 1). CD8^+^ T cell numbers were 9-fold higher than CD4^+^ T cells in PbA-infected mice. No change in neutrophils and NK cell numbers was observed (Fig. 1). Collectively, our data are consistent with previous reports showing that there is a significant increase in the number of Ly6C^hi^ monocytes [25] and CD8^+^ T cells, and to a lesser degree CD4^+^ T cells [13], [15], [21], [26] recruited to the brain during ECM.

### An improved methodology for visualizing leukocyte trafficking during ECM

We have recently established an intravital imaging model that allows us to visualize the superficial intact pial vasculature in living mice *in situ* (Fig. S3) [27]. In order to gain a better understanding of how leukocytes contribute to vascular inflammation during ECM, we adopted this approach for mice infected with PbA. The main obstacle with imaging ECM is that, often, mice may progress to severe disease within only a few hours thereby leaving a short time window for surgical preparation and intravital imaging. We therefore opted for an open skull preparation, whereby a cranial window is prepared by skull bone removal under the least possible traumatic conditions and the shortest possible time frame (∼70 min). We observed that mice that developed NS resisted anaesthetic action. To overcome this, we allocated increased anaesthesia time for infected mice (3 times longer than healthy mice). Further, mice with an NS score of <7 were largely selected for our studies because these mice tolerated anaesthesia, surgical procedure and intravital imaging. A template for anaesthesia monitoring is provided in Table S1. Our procedure was further optimized for providing lasting pain relief with buprenorphine, a non-steroidal opiate that has no known immunosuppressive function [27]. This is important as the immunosuppressive action of other anti-inflammatory drugs and analgesics potentially interferes with the development of immune responses during ECM [28]. Mice were maintained at a core body temperature of 37°C in order to obviate the introduction of artefacts during the surgical procedure and intravital imaging, as reported [27]. Core body temperature was regulated by using a heat pad connected to a rectal probe and covering mice with a thermal blanket. For uniformity in imaging conditions, mice with ECM were maintained under the same optimised conditions as healthy mice. Under these conditions, mice with ECM did not develop hypothermia, which is normally observed during the progression of disease [29].

### Leukocyte accumulation occurs in a stage dependent manner during ECM

**Figure 2:**
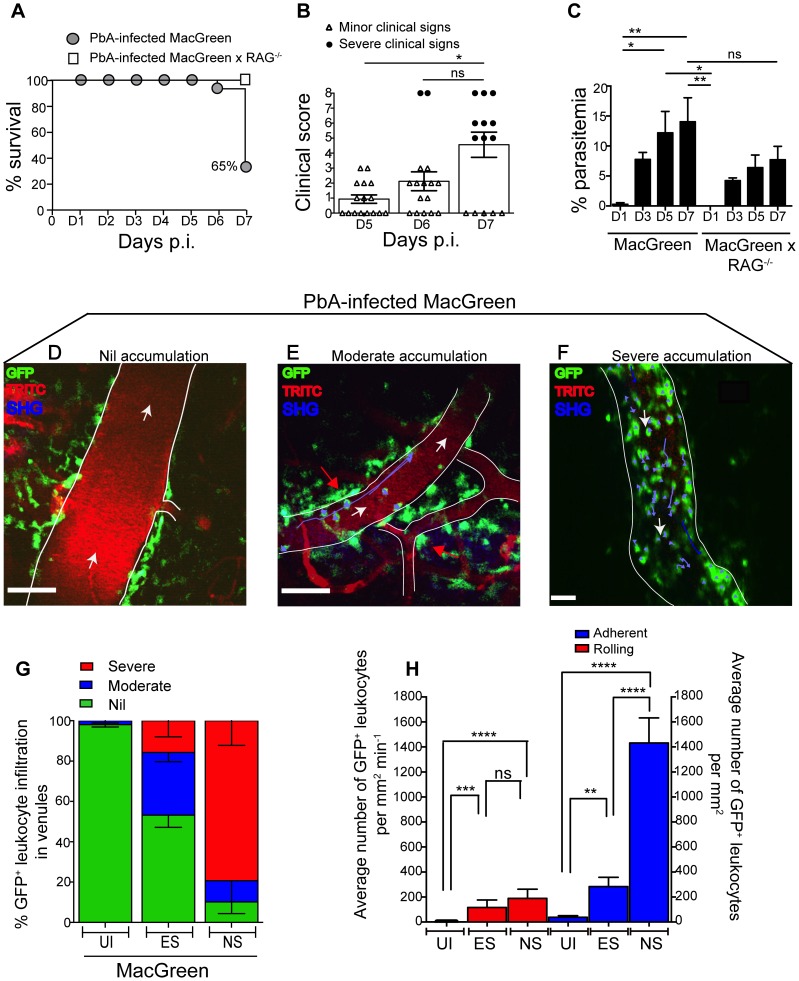
Visualizing the behaviour of GFP^+^ leukocytes in the brain microvasculature. PbA-infected MacGreen (n = 16) and MacGreen×RAG^−/−^ mice (n = 4) were monitored for (**A**) % survival to pre-defined clinical endpoint, (**B**) clinical score of each mouse on day 5–7 p.i., *p<0.05, ns, not significant (Mann-Whitney test), or (**C**) % parasitemia (n = 4 mice/group), ns, not significant, *p<0.05, **p<0.005 (One way ANOVA, Tukey's multiple comparisons test). Each symbol in (B) represents one mouse. Data are a mean of 3–6 independent experiments. MacGreen mice underwent intravital imaging of the brain microvasculature at various stages including when mice were uninfected (UI) (n = 4 mice), during ES on day 5–6 p.i. (n = 5 mice) and after they developed NS on day 7 p.i. (n = 3 mice). (**D**), (**E**), (**F**) Representative snapshots of 3–5 independent intravital imaging experiments shows nil, moderate and severe levels of leukocyte accumulation. Migratory paths of GFP^+^ leukocytes are shown as purple tracks. Blood vessels are marked by infusion of TRITC-conjugated dextran. Blue represents second harmonic generation (SHG) signals from extracellular matrix fibres. White arrows indicate direction of blood flow. Perivascular myeloid cells are indicated by red arrows. Scale bars represent 100 µm in (D), 60 µm in (E) and 28 µm in (F). (**G**) % blood vessels that have nil, moderate and severe levels of leukocyte accumulation in UI, ES and NS. (**H**) Average number of rolling and adherent leukocytes per mm^2^ of endothelium. A total 89, 90 and 43 blood vessels were assessed for UI, ES and NS groups, respectively. Number of blood vessels assessed per mouse: UI (14, 30, 28, 17), ES (14, 15, 23, 22, 16) and NS (12, 25, 6). Bars represent mean ± SEM. ns, not significant, *p<0.05, **p<0.005, ****P<0.0001 (Kruskal-Wallis-Test, Dunn's multiple comparisons test).

To determine the kinetics of vascular leukocyte accumulation in the course of ECM, we made use of MacGreen transgenic reporter mice in which GFP expression in the circulation is confined mainly to monocytes and granulocytes [30], [31]. GFP expression can also be seen in other immune cells in the brain parenchyma [30], [31] such as perivascular myeloid cells. MacGreen mice were infected with PbA and monitored for clinical signs of ECM. MacGreen×RAG^−/−^ mice, which were derived by crossing MacGreen mice with lymphocyte deficient RAG-1^−/−^ mice [32], were also infected with PbA. PbA-infected MacGreen×RAG^−/−^ mice did not develop any severe clinical signs at the pre-defined clinical end-point of day 7 p.i., as reported previously [24], [33], and served as a negative control (Fig. 2A). On the other hand, 65% of the PbA-infected MacGreen mice that had developed minor clinical signs on day 5–6 p.i. (early stage, ES) progressed on to develop severe clinical signs (NS) on day 7 p.i. (Fig. 2A, B). Mean parasitemia levels progressively increased in MacGreen mice (Fig. 2C) with levels peaking on day 7 p.i. (p<0.005). Peak parasitemia in MacGreen mice was not significantly different (p = ns) from that of MacGreen×RAG^−/−^ mice on day 7 p.i.

We then used 2P-IVM to track the behaviour of GFP^+^ leukocytes in PbA-infected MacGreen mice in the three experimental groups (UI, ES and NS). First, a baseline value of the average number of rolling and firmly adherent GFP^+^ cells in the vasculature of UI mice was determined. Rolling cells were defined as single, round-shaped endothelium-interacting cells moving in the direction of the blood flow at a lower speed than free flowing cells. In UI mice, circulating cells were mostly non-interactive, with leukocytes largely adhering at a baseline value of 0–300 cells/mm^2^ min^−1^. Next we arbitrarily graded leukocyte accumulation in the diseased animals relative to UI mice as follows: nil (0–300 cells/mm^2^ min^−1^, in the same range as UI), moderate (300–1000 cells/mm^2^ min^−1^) and severe (1000–10,000 cells/mm^2^ min^−1^) interactions. Examples of the different grades are shown in Fig. 2D–F (Movies S1, S2, S3). To assess leukocyte accumulation during ES, mice with minor clinical signs were assessed on day 5–6 p.i. We found that 31% of venules had moderate and 15% had severe levels of leukocyte accumulation during ES (Fig. 2G). With the onset of NS, leukocyte accumulation further increased with 10% of venules showing moderate and 79% showing severe levels (Fig. 2G). This was paralleled by an increase in the absolute number of endothelium-interacting GFP^+^ leukocytes in the CNS vasculature, with cells rolling along (116±60 cells/mm^2^ min^−1^) or adhering to the endothelium (283±74/mm^2^) as early as day 5–6 p.i. (Fig. 2H). With the progression of disease from ES to NS, the number of adherent leukocytes increased significantly to an average of 1432±200/mm^2^, while rolling cell numbers did not change (Fig. 2H).

### GFP^+^ leukocytes interacting with the vascular endothelium are monocytes

**Figure 3:**
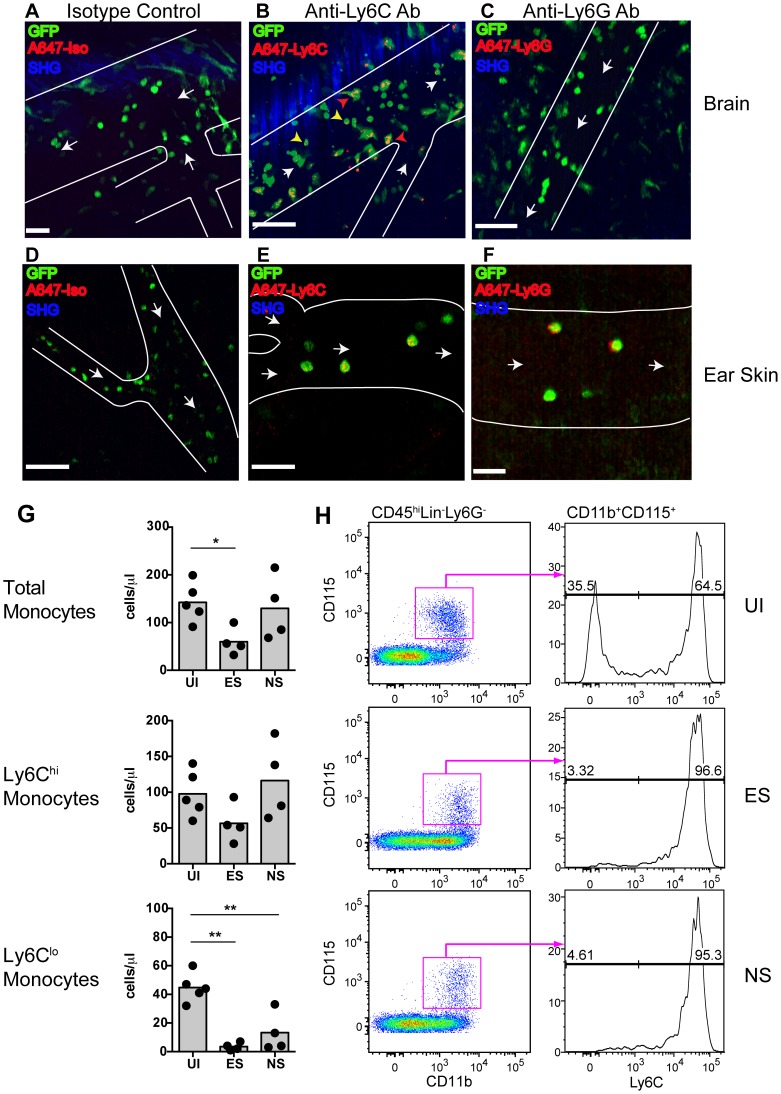
Endothelium-interacting GFP^+^ leukocytes in the brain microvasculature are monocytes. PbA-infected MacGreen mice with NS were injected i.v. with 5 µg of either (**A**) an isotype control, (**B**) anti-Ly6C (n = 2) or (**C**) anti-Ly6G antibody (n = 2) 1 hour prior to mice undergoing intravital imaging of the brain microvasculature. Representative snapshots from each group are shown. Scale bars represent 30 µm in (A), 100 µm in (B) and 58 µm in (C). Blue represents SHG signals from extracellular matrix fibres. White arrows indicate direction of blood flow. To aid orientation, vascular walls are outlined. Cells stained with Ly6C antibody show an orange punctate stain that co-localises with GFP (red arrowheads = adherent cells; yellow arrowheads = rolling cells). A total of 37, 29 and 45 blood vessels were assessed for the isotype control, Ly6C and Ly6G groups respectively. Number of blood vessels per mouse: Isotype (n = 37), Ly6C (n = 14, 15) and Ly6G (n = 9, 36). Representative snapshots of intravital imaging of the dermis of Lysozyme-GFP mice after injecting 5 µg of (**D**) an isotype control, (**E**) anti-Ly6C or (**F**) anti-Ly6G antibody, as reported [34]. Number of blood vessels per mouse: Isotype (n = 12), Ly6C (n = 11) and Ly6G (n = 11). Cells stained with Ly6C show a punctate stain as in the brain. Cells stained with Ly6G have a red surface stain. Scale bars represent 60 µm in (D), 30 µm in (E) and 20 µm in (F). (**G**) Changes in blood monocyte populations during ECM. Blood monocytes were quantified by flow cytometry after collection from either uninfected (UI) or PbA-infected mice that had developed ES or NS (n = 4–5 mice/group). Symbols represent individual animals and bars represent means. *P<0.05, **p<0.01 (ANOVA with Dunnett's post-test vs. UI control). (**H**) Representative flow cytometric plots showing blood monocyte populations in UI, ES and NS mice. The percentage of Ly6C^lo^ and Ly6C^hi^ monocytes are indicated. Data are from a single experiment (n = 4–5 mice/group).

To determine the lineage of GFP^+^ leukocytes that accumulate in the venules of MacGreen mice during NS, we administered antibodies against Ly6C or Ly6G or an isotype control antibody intravenously (i.v.) [34]. The Gr1 and Ly6C surface markers are expressed by both GFP^+^ granulocytes and monocytes, whereas Ly6G is expressed selectively by neutrophils. We found that the adherent GFP^+^ leukocytes did not stain for the isotype control (Fig. 3A) (Movie S4), but that the majority were Ly6C^+^ (Fig. 3B) (Movie S5). Several rolling leukocytes also stained for Ly6C. Stationary Ly6G^+^ cells were not seen (Fig. 3C) (Movie S6). We validated the used antibodies also in an ear skin-imaging model in Lysozyme-GFP mice, where GFP^+^ neutrophils are known to roll along non-inflamed dermal post-capillary venules [34], [35], unlike the non-inflamed pial vasculature where such events are almost entirely absent [36]. While no staining was observed with the isotype control (Fig. 3D) (Movie S7), GFP^+^ cells were positive for both Ly6C (Fig. 3E) (Movie S8) and Ly6G (Fig. 3F) (Movie S9). Further confirmation of the identity of adherent GFP^+^ cells was obtained with antibody staining of brain sections (Fig. S4). Thus, along with the flow cytometric data presented in Fig. 1, these results show that the adherent GFP^+^ cells in the CNS vasculature during ECM are Ly6C^hi^ monocytes.

Concomitantly with increased monocyte adherence, we observed a transient decrease in total circulating monocyte numbers during ES, which was restored during the later NS (Fig. 3G). Notably, Ly6C^lo^ monocytes largely disappeared from the circulation, corresponding with a shift towards a Ly6C^hi^ subset (Fig. 3G, H). Our data show that there is an overall switch to Ly6C^hi^ monocytes within circulation during infection, and this translates to increased adherence to the vascular endothelium of the brain during ECM.

### Quantification of monocyte rolling and adherence in the course of ECM

As another measure of endothelial cell activation during ECM development, we measured rolling velocities of GFP^+^ cells in MacGreen mice. As reported, GFP^+^ cells were largely non-interactive in UI animals [36], whereas during ES they were observed rolling along the endothelium (Fig. S5A; V_mean_: 22.9±1.3 µm/sec); which is within the range of rolling velocities described for leukocytes in other vascular beds (20–60 µm/sec) [37]). During NS, the majority of the GFP^+^ cells became firmly adherent, with the remaining rolling cells slowing down considerably (V_mean_: 6.3±1.8 µm/sec; Fig. S5B). Three representative tracks that depict the instantaneous velocities of monocytes during ES and NS are shown in Fig. S5C and D. The average velocity of rolling monocytes in severely inflamed venules (V_mean_: 4.7±1.2 µm/sec) was significantly lower than that in moderately inflamed venules (V_mean_: 23.0±1.3 µm/sec) (Fig. S5E).

Studies have shown that myelomonocytic cell recruitment by CTL and their extravasation leading to CNS injury plays an important role in viral infection [38]. Therefore we asked whether monocytes also extravasated during ECM. We did not observe extravasation events of rolling/adhering monocytes during the observation period of up to 1.5 hours (Fig. S6A) (Movie 10). GFP^+^ cells typically travelled in the same direction as that of the blood flow (Fig. S6B) and exhibited very little deviation from their path during ES or NS as evident from the meandering index [MI] [ES: 0.92±0.01] [NS: 0.87±0.02] (Fig. S6C). To further immunophenotype GFP^+^ cells residing in the perivascular space of UI and PbA-infected MacGreen and MacGreen×RAG^−/−^ mice, we used confocal microscopy (Panel i–iii, Fig. S6D). We injected wheat germ agglutinin (WGA) conjugated to Alexa 594 to mark circulating monocytes [39], and then prepared sections of the brain. Round GFP^+^ WGA^+^ intravascular monocytes were mostly F4/80^−/lo^ (yellow overlay) whereas stellate-shaped GFP^+^ perivascular cells were mostly F4/80^+^ (blue-green overlay). We concluded that monocytes by and large do not extravasate in the course of ECM, which is consistent with our previous histopathological studies [3], [40]. GFP^+^ cells in the perivascular space most likely represent perivascular macrophages in MacGreen mice [41], [42].

### Primed CD8^+^ T cells induce NS in immunodeficient RAG^−/−^ mice and promote high levels of monocyte accumulation in the brain microvasculature

**Figure 4:**
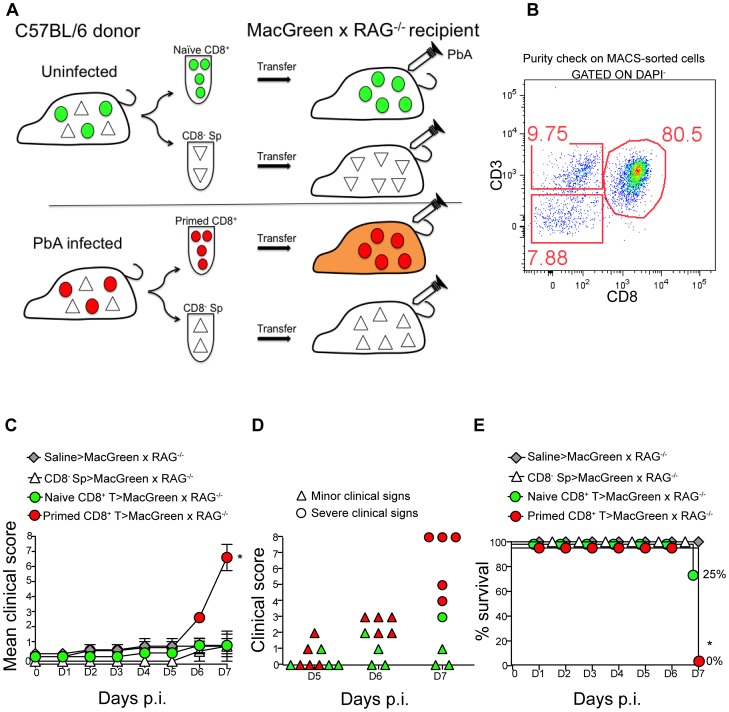
An adoptive transfer model to study the regulation of monocytes during ECM. (**A**) CD8^+^ T cells or CD8^−^ splenocyte fraction (Sp) isolated by MACS from uninfected or PbA-infected C57BL/6 donor mice on day 7 p.i. were adoptively transferred into PbA-infected MacGreen×RAG^−/−^ recipient mice as depicted. Only primed CD8^+^ T cells (red circle) isolated from PbA-infected C57BL/6 donor mice induce NS (orange recipient). Representative data of 3 independent experiments is shown. (**B**) Dot plot shows purity of CD8^+^ T cells routinely obtained by MACS sorting. (**C**) MacGreen×RAG^−/−^ recipient mice receiving saline (n = 4 mice), naïve (n = 4 mice) or primed CD8^+^ T cells (n = 5 mice) or CD8^−^ splenocytes (n = 4 mice) were infected with PbA and clinical scores monitored daily, *p = 0.01 (unpaired *t* test). (**D**) Clinical score of each mouse on day 5–7 p.i. Each symbol represents one mouse. Recipients of naïve and primed CD8^+^ T cells are shown in green and red respectively. (**E**) Percent survival of MacGreen×RAG^−/−^ recipients after adoptive transfer, *p<0.05 (Mantel-Cox test).

The role of CD8^+^ T cells in promoting CM is well established [13], [15], [21], [24], [43], [44]. Therefore, we investigated whether CD8^+^ T cells regulate monocyte accumulation in mice during ECM. To this end, MacGreen and MacGreen×RAG^−/−^ mice were infected with PbA or left uninfected. In MacGreen×RAG^−/−^ mice, approximately 21% of the venules had moderate and 8% had severe levels of monocyte accumulation (Fig. S7A, B), with the total numbers of endothelium-interacting monocytes averaging 332 (±74.5)/mm^2^ min^−1^ as compared to MacGreen mice with 1432 (±200)/mm^2^ min^−1^ interacting cells (Fig. S7C, p<0.0001). To directly examine the effects of T lymphocyte subsets on ECM development, we adoptively transferred enriched CD8^+^ (80%) or CD8^−^ splenocytes isolated from UI or PbA-infected C57BL/6 donor mice into MacGreen RAG^−/−^ recipients (Fig. 4A, B). When MacGreen×RAG^−/−^ recipient mice were infected with PbA (Fig. 4A), we found that CD8^+^ T cells from PbA-infected donor mice induced severe clinical signs in all animals (Fig. 4C, D, E). Recipients of primed CD8^+^ T cells had a higher mean clinical score compared to all other groups, at least during the observation period of 7 days (Fig. 4C). Uninfected recipients that received primed CD8^+^ T cells did not develop ECM (data not shown). Of recipients receiving naïve T cells, 25% developed severe clinical signs, whereas all other groups of recipients were resistant (Fig. 4D, E).

**Figure 5:**
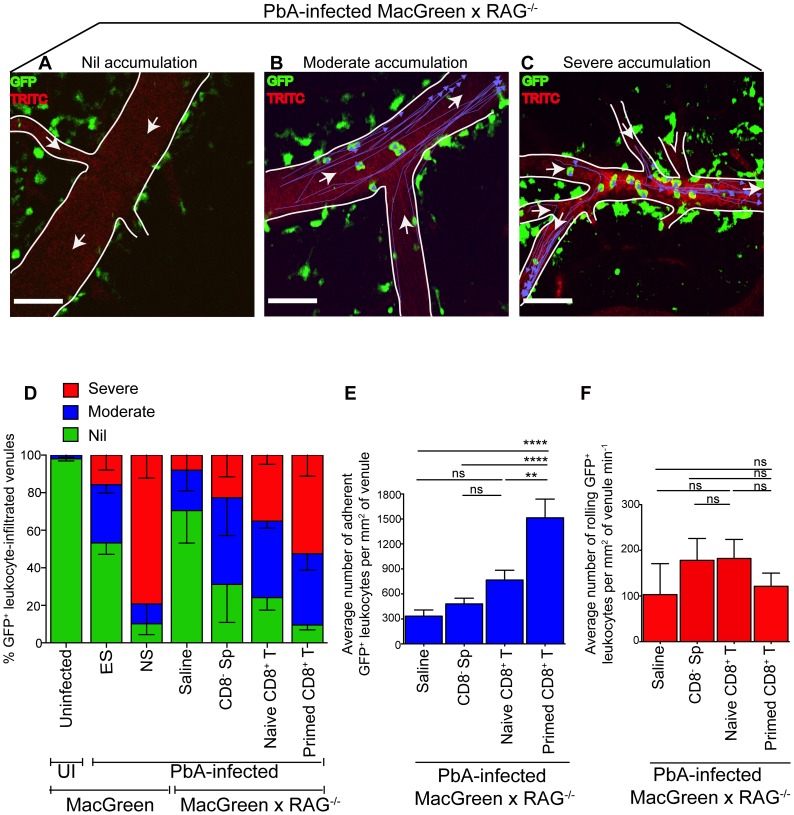
*Plasmodium*-primed CD8^+^ T cells induce monocyte accumulation in MacGreen×RAG^−/−^ mice. PbA-infected MacGreen×RAG^−/−^ mice that had received saline, CD8^−^ splenocytes, naïve or primed CD8^+^ T cells as in Fig. 4A underwent intravital imaging on day 7 p.i. (n = 3 mice/group). Representative snapshots show (**A**) nil, (**B**) moderate and (**C**) severe levels of monocyte accumulation in the blood vessels. Scale bars represent 60 µm. Migratory paths of monocytes are shown as purple tracks. Blood vessels are marked by infusion of TRITC-conjugated dextran. White arrows indicate direction of blood flow. (**D**) % blood vessels with nil, moderate and severe levels of monocyte accumulation in MacGreen×RAG^−/−^ and MacGreen mice. Average number of (**E**) adherent and (**F**) rolling leukocytes per mm^2^ of endothelium. A total 46, 123, 82 and 104 blood vessels were assessed for recipients of saline, CD8^−^ splenocytes, naïve and primed CD8^+^ T cells respectively. Number of vessels assessed per mouse: saline (n = 10, 12, 24), CD8^−^ splenocytes (n = 45, 28, 25), naive CD8^+^ T cells (n = 60, 11, 11), primed CD8^+^ T cells (n = 49, 33, 22). Blood vessel numbers for MacGreen mice are as in Fig. 2. Data are a mean of 2–3 independent experiments. Bars represent mean ± SEM, ns, not significant, *p<0.05, **p<0.001, ***P = 0.0001 (ANOVA, Tukey's multiple comparisons test).

To determine the effects of T cells on monocyte behaviour within the brain vasculature, we performed 2P-IVM on groups of MacGreen×RAG^−/−^ mice that had received saline, CD8^−^ splenocytes, naive CD8^+^ T cells or primed CD8^+^ T cells (Fig. 4A). The four recipient groups were infected with PbA and blood vessels assessed by intravital imaging on day 7 p.i. As in wildtype MacGreen mice, several degrees of monocyte accumulation could be observed in the various groups of MacGreen×RAG^−/−^ recipient mice (representative examples of nil, moderate and severe accumulation are shown in (Fig. 5A–C) (Movie S11, S12, S13). As expected, saline infusion alone resulted in low levels of inflammation (Fig. 5D). Adoptive transfer of CD8^−^ splenocytes from PbA-infected recipients resulted in an average 46% and 22% of venules showing moderate and severe levels of GFP^+^ cell accumulation, respectively. Transfer of naïve CD8^+^ T cells resulted in 40% of venules showing moderate, and 35% showing severe levels of GFP^+^ cell adhesion. Primed CD8^+^ T cells induced an average of 37% of venules showing moderate and an average of 52% showing severe levels of monocyte accumulation. In terms of absolute numbers of GFP^+^ cells adhering to the vessel wall, saline alone or CD8^−^ splenocytes only induced low levels of monocyte accumulation (332±74/mm^2^ and 479±68/mm^2^ respectively) (Fig. 5E). Adoptive transfer of naïve CD8^+^ T cells did not change the phenotype significantly from that induced by saline or CD8^−^ splenocytes with monocytes adhering to the endothelium at 767±117.7/mm^2^. On the other hand, primed CD8^+^ T cells led to significantly more monocytes adhering within the vasculature compared to groups receiving other treatments, at an average of 1513±225.2/mm^2^ (Fig. 5E). Mice with this phenotype developed NS. Rolling cell numbers did not change between the three groups (Fig. 5F). Transfer of primed CD8^+^ T cells caused monocytes to slow down considerably as compared to naïve CD8^+^ T cells (not shown). The average velocity of rolling monocytes in severely inflamed venules (V_mean_: 18.9±1.03 µm/sec) was lower than in moderately inflamed venules (V_mean_: 29.9±2.2 µm/sec) (Fig. S8). Collectively, our data show that the clinical signs of NS induced by primed CD8^+^ T cells are associated with high levels of monocyte accumulation in venules as well as slowing down of the rolling monocytes.

Next, to determine the critical number of primed effector CTL that are required to induce monocyte adhesion and disease, we performed dose titration experiments. Primed CD8^+^ T cells (10, 4.5, 3 and 0×10^6^) were transferred into PbA-infected MacGreen×RAG^−/−^ recipients and on day 7 p.i., the blood vessels in each of the four groups were assessed by intravital imaging. Mice that received 4.5, 3 and 0×10^6^ T cells did not develop NS (Fig. S9A) with approximately 23% and 50% of venules lacking monocyte accumulation in mice that received 4.5×10^6^ and 3×10^6^ T cells respectively (Fig. S9B). In contrast, 10×10^6^ CTL consistently induced NS, with only 11% venules lacking monocyte accumulation. Of interest, recipients of 10×10^6^ naïve CD8^+^ T cells lacked monocyte accumulation in 25% of venules and were also protected from NS (Fig. 5D). Furthermore, transfer of 40×10^6^ naïve CD8^+^ T cells into PbA-infected MacGreen×RAG^−/−^ mice failed to increase monocyte accumulation or induce NS (not shown). Taken together, these data show that a critical number of primed CD8^+^ T cells is required in order to induce high levels of monocyte adhesion and clinical disease.

### Monocyte depletion decreases CD8^+^ T cell recruitment but does not alter disease course

**Figure 6:**
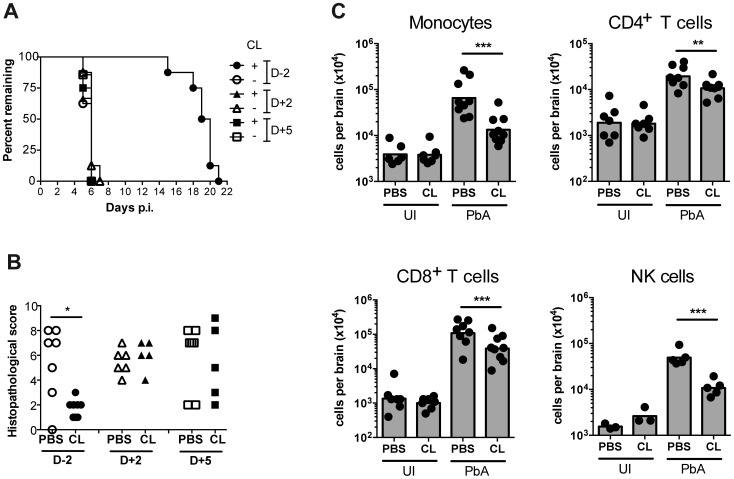
Effect of monocyte depletion on the development of ECM. (**A**) Mice were infected with PbA and administered with CL or sham-treated (PBS) i.v., at the indicated times. Survival time to a predetermined clinical endpoint is shown. (**B**) Histopathological scores of brain sections from mice in panel A. *p<0.05 (Mann-Whitney test vs. PBS control). (**C**) Effect of CL treatment on brain leukocyte populations. Uninfected (UI) or infected (PbA) mice were treated with CL or PBS on day +5 p.i., sacrificed on developing ECM (day 6), and the indicated BSL populations were quantified by flow cytometry. Symbols represent individual animals and bars the geometric mean. **P<0.01, ***p<0.001 (ANOVA on log-transformed data with Tukey's post-test). Data are pooled from two independent experiments (A, C) or a single experiment (B). NK cell data are from a single experiment.

Given the observation of inflammatory monocyte recruitment to the brain during ECM, we investigated their role in the development of neuropathology. Circulating monocytes, and other phagocytic populations that are in contact with the peripheral blood, were depleted by i.v. injection of clodronate liposomes (CL) [45] either at two days prior to infection (day −2), two days p.i. (day +2), or 5 days (day +5) p.i. Administration of CL prior to infection led to complete protection from ECM, with mice ultimately succumbing to high parasitemia and low haematocrit (Fig. 6A, 6B, S10A, S10B). There was no difference in disease course between PbA-infected mice that were depleted of monocytes at day +2 or day +5 p.i. when compared to vehicle treated mice (Fig. 6A). Nevertheless, mice that received CL late in the disease course (day +5) showed decreased recruitment of BSL compared to sham-depleted controls. As expected, treatment with CL resulted in an 80% decrease in brain-recruited monocytes following CL treatment (Fig. 6C). In addition, CD4^+^ and CD8^+^ T cells and NK cell numbers were also decreased by 1.8-fold, 2.8-fold and 4.6-fold, respectively, compared to sham-treated control animals, indicating the role of monocytes/macrophages in lymphocyte recruitment to infected brain during ECM.

## Discussion

Discussion on the type of leukocytes that sequester inside the blood vasculature has gained significance due to discrepancies that have been reported between the pathological features of the ECM model and human CM [46]. A recent report tries to look beyond the differences in the cell type that sequester to the endothelium during human CM and ECM [47]. It attempts to unify the two by suggesting that the outcome of the neuropathological syndrome may be similar (i.e. impaired blood flow, altered hemodynamics and tissue necrosis). A consensus has since emerged within the field that experimental studies in the mouse model of ECM must be directed towards understanding the pathological processes within the brain that lead to NS and the relevance of these localised events to human CM. As a step in this direction, we performed real-time imaging of leukocyte behaviour in the CNS microvasculature in order to understand the role of leukocytes in ECM development.

To achieve this, we optimised a brain-imaging model specifically designed for imaging mice with ECM [27]. Using this model we assessed the dynamics of the leukocyte responses during ECM in the absence of anti-inflammatory drugs. Another recent report has utilized a chronic cranial window approach generated in healthy mice 1–2 weeks before they develop ECM [48]. Anti-inflammatory drugs were used to suppress inflammation induced by the cranial window preparation on the basis that drug effects will subside during the recovery period prior to imaging. Nevertheless, anti-inflammatory drugs can potentially impact on immune responses during the early stages of disease, given the difficulties of maintaining a chronic cranial window in a drug-free environment [49], [50]. Another factor that significantly impacted on the quality of intravital images was the anaesthetic state of mice. Undoubtedly, intravital imaging studies in the ECM model have been hampered until now due to technical difficulties [51] such as anesthetising mice prior to surgery. To overcome this, a majority of our recordings were acquired when mice were most responsive to anaesthesia (mild clinical signs of ES on day 5–6 p.i. or <7 severe clinical signs of NS on day 7 p.i.). This strategy increased the survival rate of mice during and after surgery and obviated tracking cells within an immunosuppressed microenvironment.

Over the past decade, the role of CD8^+^ T cells in driving NS has become well established, however the other populations of leukocytes sequestering within the vasculature have not been adequately characterised. Macrophages, neutrophils, CD4^+^ and CD8^+^ T cells have all been reported to increase in numbers during ECM [14], [15]. While using T cell-deficient mice or depleting CD8^+^ T cells in mice abrogated clinical signs and neuropathology [13], [19], [20], [52], depleting F4/80^+^ macrophages (with clodronate liposomes) and antibody-mediated depletion of neutrophils in the late stages of ECM failed to confer protection from NS [14], [15]. This was further demonstrated using the MAFIA mice wherein conditional ablation of about 80% of the total CD115^+^ myeloid cells did not affect the parasite biomass in the head or prevent ECM [53]. Similarly, depleting brain-sequestered FcεRI^+^ granulocytes from day 5 p.i. did not protect mice from ECM although this population was reported to play a crucial role in the lead up to disease [54]. The above studies are contradicted by an earlier report showing that BSL are not granulocytes but Ly6C^+^ monocytes, which also sequester to the endothelium of PbA-infected mice that do not develop ECM [55], [56]. Although no clear functional role has been attributed to these leukocytes in inducing NS, one study found that adherent leukocytes reduce the luminal diameter of the blood vessels and function as barriers to blood flow during ECM [57]. This is a reminder that functional studies can sometimes be limited by a requirement for sensitive cellular and imaging tools to help delineate complex mechanisms that contribute to clinical disease. We therefore undertook a comprehensive phenotypic and functional analysis of leukocytes in the CNS vasculature using multiple technical approaches. Our studies show that the majority of the BSL are Ly6C^hi^ monocytes or CD8^+^ T cells as assessed by flow cytometry with no increase in granulocyte numbers. Thus, the molecules that promoted myeloid cell adhesion to the vascular endothelium appear to be monocyte-specific since neutrophils did not adhere, consistent with previous reports [55]. Using intravital imaging, we further confirmed that adherent leukocytes were Ly6C^+^Ly6G^−^ monocytes. Confocal microscopy of whole mount brain sections revealed that the intraluminal monocytes not only accumulated within the superficial pial vessels but also in the deeper vessels of the cerebral cortex (not shown).

Studies have proposed that monocytes can transform into macrophage-like antigen presenting cells within the blood vessels and engage in intravascular antigen presentation to T cells [58]. Our data show that, during ECM, F4/80^−/lo^ monocytes remain mostly single, round, discrete, non-vacuolated cells that can be clearly distinguished from the stellate morphology of F4/80^+^ vacuolated, perivascular myeloid cells that flank the blood vessels [30], [31]. We are unable to formally exclude the possibility of perivascular myeloid cells originating from extravasated monocytes that underwent differentiation; however this is unlikely as perivascular myeloid cells are detected in uninfected MacGreen mice and their numbers did not increase significantly following PbA-infection. Further, it is also unlikely that, during NS, all monocytes that extravasate undergo differentiation into perivascular myeloid cells within a short span of only a few hours. Historically, leukocyte extravasation has not been documented in the ECM model and our histological studies support these observations [3], [40].

We observed that monocytes begin interacting with the endothelium as early as day 5–6 p.i. when mice have only minor clinical signs of ES. The factors that modulate the vascular endothelium during ECM could thus be produced far earlier than envisaged. As the disease progressed, we saw a shift in behaviour with increasing numbers of monocytes adhering to the endothelium of post-capillary venules. This was accompanied by a significant decrease in the velocity of rolling monocytes. Vascular obstruction was unlikely to be a reason for the decreased velocity as we assessed only blood vessels that supported high velocity, free flowing cells [57]. In support of our view, recent reports have shown that a decrease in the velocity of circulating pRBC during ECM is rarely accompanied by vascular occlusion [59]. However, it should be pointed out that we did not directly correlate red blood cell (RBC) velocities and rolling behaviour of monocytes. Thus it is conceivable that in severely inflamed blood vessels a decrease in blood flow may have contributed to the observed reduction in the velocity of rolling monocytes [59].

To understand the cellular mechanism that regulates monocyte behaviour, we set up an adoptive transfer model. Primed CD8^+^ T cells transferred from PbA-infected mice were mostly differentiated effector cells (data not shown and [13], [52]) that had a T cell repertoire specific to *Plasmodium* antigen. We found that, following adoptive transfer, CD8^+^ T cells promoted monocyte adhesion to the vascular endothelium and led to a significant decrease in the rolling velocity of monocytes. Primed T cells were more effective than CD8^−^ splenocytes or naïve T cells in promoting monocyte adhesion, slowing down rolling monocytes and inducing disease and this coincided with a significant number of primed CD8^+^ T effector cells being recruited to the brain (not shown). These data are in line with reports of CD8^+^ T cell recruitment to and activation of CTL function in the CNS vasculature leading to inflammation and disease [16], [21], [24], [60], [61]. Further, primed T cells induced ECM in MacGreen×RAG^−/−^ recipient mice within the same time frame as wild type MacGreen mice suggesting that primed T cells, unlike naïve T cells, can be recruited directly to the brain while bypassing the requirement for priming. Furthermore, uninfected recipients that received primed CD8^+^ T cells did not develop ECM (data not shown), suggesting a requirement for *Plasmodium* antigen at the effector phase, as reported [21], [24], [62].

While the recruitment of CD8^+^ T cells to the brain during ECM is dependent upon IFNγ driven production of the CXCR3-binding chemokines CXCL9 and CXCL10 [16], [44], [60], the origin of this IFNγ is debated. Either NK cells [63] or CD4^+^ T cells [64], [65] have been proposed to be the cellular source of this critical IFNγ. In our hands, transfer of purified primed CD8^+^ T cells into infected RAG-1^−/−^ recipients (which retain NK but lack CD4^+^ T cells) resulted in the development of ECM. These data echo those of Nitcheu et al., who transferred CD8^+^ T cells of >98% purity into RAG-2^−/−^ mice and demonstrated the capacity of CD8^+^ T cells to drive neuropathology and disease in the absence of CD4^+^ T cells [13]. Similarly, we found that transfer of OT-I effector cells (>98% purity) into RAG^−/−^ recipient mice infected with OVA-transgenic parasites (PbTG) led to disease, as reported (data not shown and [24]). Nevertheless a small number of CD4^+^ T cells were recruited to the brain of mice that developed NS (primed CD8^+^ T cell recipients) as well as in mice that were protected from disease (CD8^−^ splenocyte recipients) therefore we can not formally exclude that small numbers of CD4^+^ T cells contributed to CD8^+^ T cell recruitment.

Although some studies have suggested that monocytes are essential for the induction of ECM [66], [67], others have argued that these cells are dispensable [68]. We addressed the role of monocytes by administration of CL. Since CL administration does not result in complete depletion, unequivocal conclusions about the functional role of these cells could not be drawn at this stage; however our data did reveal an influence of phagocytic cells, including monocytes, in both early and late processes underlying ECM. Depletion prior to infection resulted in diversion of the disease course away from cerebral pathology. This observation is consistent with involvement of a phagocytic population in induction of immune processes that normally culminate in ECM. The most likely phagocytic population is Clec-9a^+^ DCs that are responsible for cross-presentation of *Plasmodium* antigens to CD8^+^ T cells [69]. When monocytes were depleted by CL administration late in the course of infection, there were significant decreases in sequestered CD8^+^ T, as well as CD4^+^ T and NK cells within the brain compared to sham depleted controls. Consistent with monocyte contribution to this process, we have previously shown that, during ECM, cells sequestered within the vasculature express CXCL9 mRNA [44]. Nevertheless, monocyte depletion did not alter the course of disease progression. This phenomenon is potentially explained by results from our adoptive transfer model where we found that recruitment of a very small number (∼35,000) of CD8^+^ T cells recruited to the brain is sufficient to induce neuropathology (not shown). Thus although monocytes contribute to CD8^+^ T cell recruitment, in their absence sufficient T cells are still recruited to the brain to initiate ECM.

Although monocytes themselves were not essential to ECM pathology, their recruitment to the brain vasculature appeared to be a sensitive readout that correlated with end stage pathological processes. Our adoptive transfer model enabled us to track precisely how primed CD8^+^ T cells regulated monocyte trafficking in the microvasculature of the brain during disease. We found that even a small increase in the numbers of severely inflamed venules (17% increase in mice that received primed T cells as compared to naive T cells) was associated with a strikingly different clinical outcome (Fig. 5D). Thus monocyte accumulation served as a useful surrogate marker for visualising the unfolding of vascular inflammation in the CNS. Altogether our data support the view that NS manifestation correlates with CD8^+^ T effector cells promoting a critical threshold of monocyte accumulation in the venules. To this end, a critical number of CD8^+^ T effector cells were required to attain this threshold. The underlying molecular mechanism/s by which CD8^+^ T cells promote monocyte accumulation are unclear; however specific blockade of LIGHT-LTβR interactions during PbA infection was shown to decrease monocyte sequestration in the brain [70]; a potential role for LIGHT or LT-alpha/beta expressed on activated CD8^+^ T cells [71] can therefore be envisaged in facilitating monocyte sequestration. Similarly, IFNγ along with TNF and LT-alpha is a candidate cytokine that can promote monocyte sequestration by increasing the expression of adhesion molecules such as ICAM-1, VCAM-1, P-selectin and E-selectin on mouse brain endothelial cells [72]. Altogether, our observations are consistent with a model in which damage to the blood-brain barrier (BBB) and vascular leakage caused by CD8^+^ T cell-mediated cellular/molecular factors is ultimately responsible for disease and death during ECM [59], [73].

## Materials and Methods

### Ethics statement

This study was carried out in accordance with the guidelines from The National Health and Medical Research Council of Australia. All animal procedures were carried out under the protocol number K75/11-2009/3/5195, approved by the University of Sydney Animal Ethics Committee. All mice were maintained with environmental enrichment in specific pathogen-free (SPF) conditions at the Centenary Institute Animal Facility. The surgical procedures and intravital imaging experiments were carefully optimised [27] and performed by trained specialists. The anaesthetic drugs Ketamine, Xylazine and Buprenorphine were used for anaesthetising mice prior to surgery.

### Mice

All mice used in this study were on a C57BL/6 background. Lysozyme-GFP mice [74], and *c-fms*-GFP (MacGreen) mice [30], [31] were described previously. C57BL/6 and RAG-1^−/−^ mice were obtained from the Australian Resource Centre (ARC) [75]. Wildtype MacGreen^+/+^ were outcrossed with RAG-1^−/−^ mice to derive F1 MacGreen×RAG^+/−^ heterozygous mice. Heterozygous parents were intercrossed to derive MacGreen×RAG^−/−^ homozygotes. Litters underwent prior screening for GFP and CD3 (Fig. S11A) as well as B220 expression by flow cytometry and for RAG-1 gene expression by PCR (Fig. S11B). Only CD3^−^ GFP^hi^ MacGreen×RAG^−/−^ mice were used in this study.

### Parasites and infections

PbA strain was maintained and used as previously reported [76]. All wildtype, transgenic and knockout mice were infected intraperitoneally (i.p.) with 10^6^ pRBC. Parasitemia was determined by thin blood smears prepared from tail bleeds. Smears were stained using the Diff-Quick kit (Lab Aids, Narrabeen, NSW, Australia).

### Clinical disease assessment

PbA-infected MacGreen mice that developed minor clinical signs were referred to as being in the early stage or ES (Fig. S1). ES was defined by reduced activity, hair standing on end, prominent vertebral spinous processes/scapulae/pelvis, sunken eyes and/or hunched posture. Each minor clinical sign was given a score of 1 with mice receiving a maximum ES score of 2. Mice with ≥3 minor clinical signs on days 1–6 p.i. were euthanized in accordance with ethics guidelines and given a maximum score of 8 the following day to denote death. Mice were allowed to develop severe clinical signs on day 7 p.i. defined by ataxia, immobility, isolation from group, reduced searching behaviour, muscular weakness, hemiparesis, paraparesis, inability to grab the cage grid and/or hold on to a rope, fitting, inability to roll and/or spasmodic backbend (Fig. S1). These mice were referred to as having neurological signs or NS. Each severe clinical sign was given a score of 1 with mice receiving a maximum NS score of 8. All mice in the experimental study were sacrificed at the pre-defined clinical end-point of day 7 p.i. A modified clinical scoring system was used for monocyte depletion experiments, with animals being euthanized if they manifested with any of the following signs: decreased stimulated movement with unsteady gait, hemiplegia, seizures or inability to grab the cage grid.

### Antibodies and reagents

Fluorochrome-labelled antibodies were purchased from BD Biosciences and Biolegend and included anti-CD3, CD4, CD8, CD11b, CD19, CD45, CD335, NK1.1, B220, F4/80, Ly6C and Ly6G (Table S2). For *in vivo* labelling, WGA-A594 (Life Technologies) was administered i.v. in mice just prior to sacrifice. Mice were perfused intracardially and brains were harvested. Similarly one hour prior to intravital imaging, 5 µg of anti-Ly6C, Ly6G or isotype antibody (Biolegend) was administered i.v. in mice via the lateral tail vein to label cells *in vivo*. CL containing 5 mg/ml clodronate were purchased from www.clodronateliposomes.com. Monocytes were depleted by a single i.v. injection of 200 µl of CL on the day indicated in the main text.

### Sample preparation for flow cytometry

To purify leukocytes from the brain, mice were sacrificed and perfused via the heart with 20 ml of perfusion fluid (0.05% w/v EDTA in ice cold PBS) to remove blood contaminants and non-sequestered cells from the cerebral vasculature. Brains were initially mashed between frosted glass slides in PBS. Subsequently, they were digested with collagenase type IV (Sigma) and DNase I (Sigma) for 20 min at room temperature, and triturated through a Pasteur pipette before a second 20 min collagenase/DNase incubation. Low-density dead cells/debris/myelin were removed by resuspension in 30% Percoll and centrifugation at 500 g for 10 min at 4°C. Contaminating RBCs were then lysed using Tris-ammonium chloride buffer and leukocytes were washed and enumerated. To process brains harvested from RAG^−/−^ mice, whole brains weighing up to 600 mg were placed in 2650 µl of PBS containing 0.05% v/v FCS, collagenase type IV (2 mg/ml) and DNAse I (0.5 mg/ml). For brains weighing more than 600 mg, suspension volume was scaled up accordingly. Brains were homogenized using a gentleMACS dissociator (Miltenyi). Tissue suspension was digested by rotational mixing at 37°C for 60 min and, after a wash, debris/myelin were removed by Percoll centrifugation as above. For flow cytometry on blood leukocyte populations, 20 µl of blood was collected from the tail using EDTA as an anticoagulant and red blood cells lysed using Tris-ammonium chloride buffer. Following lysis, cells were washed and stained as described below.

### Flow cytometric analysis

Cells obtained from the brain were suspended in 2% (v/v) FCS in PBS (FACS wash buffer containing 2 mM EDTA and 0.01% w/v sodium azide) containing anti-CD16/32 (2.4G2) (BD) to block Fc receptors. Cells were stained by incubating with fluorochrome-labelled antibodies diluted in FACS buffer for 30 min on ice. Thereafter, cells were washed and stained with 0.5 µg/ml DAPI (Invitrogen) for dead cell exclusion. For blood leukocyte counts, samples were spiked with a known concentration of Accucount polystyrene microspheres (Spherotech, Lake Forest, Illinois) to determine absolute numbers of cells in the original sample. Data were acquired on a FACS Canto II (BD), LSRFortessa (BD) or LSR-II flow cytometer (BD) and analysed using FlowJo software (Treestar).

### T cell isolation

All cell isolations were performed in a biological safety cabinet under sterile conditions to facilitate transfer into immunodeficient mice. CD8^+^ T cells were positively selected from disaggregated splenocytes using CD8-conjugated immunomagnetic beads (Miltenyi) and MACS columns (Miltenyi). Positive fractions were further enriched by passing cells through a second MACS column as suggested by the manufacturer. Approximately 80% of the cells in the positive fraction were enriched for CD3^+^CD8^+^ T cells as assessed by flow cytometry. Approximately 5% of the CD8^−^ splenocytes constituted CD3^+^CD8^+^ T cells that had eluted into the negative fraction.

### Adoptive T cell transfer

MacGreen×RAG^−/−^ recipient mice aged 5–8 weeks were first infected with 10^6^ pRBC via the i.p. route. CD8^+^ T cells isolated by MACS positive selection and CD8^−^ splenocytes constituting the negative fraction were adoptively transferred into recipient mice 4–6 hours later. Either 3×10^6^, 4.5×10^6^ or 10^7^ cells were administered into the tail vein. PbA-infected mice were monitored as above.

### Immunohistochemistry

Mice were administered WGA-A594 i.v., just prior to sacrifice. Vascular perfusion was performed as above using 10% neutral buffered formalin (Sigma) in PBS. Brains were harvested and further fixed in 10% neutral buffered formalin and 4% w/v sucrose for 16 hours at 4°C. Longitudinal or superior transverse sections of the brain were prepared using a vibratome and then blocked in 10% FCS. Immunohistochemistry was performed by treating brain sections with anti-Ly6C, anti-Ly6G or anti-F4/80 antibody. For some sections, blood vessels were stained with purified anti-CD31 antibody, which was conjugated to Alexa594 using the monoclonal antibody labelling kit (Life Technologies). Both WGA-A594 and CD31 delineated the blood vessels. Sections were mounted in mounting medium (DAKO) and a series of single Z-stack images were acquired by confocal microscopy using a 20× dry or 63× oil objective (Leica SP5). Image analysis was done using Volocity (Perkin-Elmer).

### Histopathological scoring

The presence of typical histopathological features of ECM was determined in paraffin embedded haematoxylin and eosin stained brain sections. Slides were scored in a blinded manner for 3 features: (1) the presence of sequestered cells in the vasculature, (2) tissue oedema and (3) haemorrhages. Each feature was scored from 0–3: 0 = not present; 1 = isolated (1–2) occurrences throughout a single brain section; 2 = 2–4 occurrences; 3 = >4 occurrences. Values for each feature were added to provide a single histopathological score.

### Cranial window preparation

Mice were prepared for intravital imaging of the brain as previously described [27]. Briefly, animals were anaesthetised by i.p. injection of Ketamine (100 mg/kg of body weight) (Cenvet) and Xylazine (10 mg/kg of body weight) (Cenvet). Buprenorphine (Cenvet) was administered at 100 µg/kg of body weight i.p. for lasting pain relief. Animals were monitored for awareness signs such as whisker twitching, palpebral (blink) reflex, pedal withdrawal reflex and respiration rate, and surgical procedures were initiated only after the animal entered a deep state of anaesthesia (Table S1). A primary dose of Ketamine/Xylazine mixture was given increased time for anaesthetic action (3 times longer than healthy mice) and a booster dose was administered only after this time. Importantly, anaesthetic strategy was individually tailored to each mouse—for example, mice with paraparesis (partial paralysis in the lower limbs) were expected to take 3 times as long as healthy mice to lose their pedal-withdrawal reflex. Loss or regain of reflexes was closely monitored throughout the procedure and booster doses of Ketamine and Xylazine were administered as required.

Anaestethized mice were placed on a heat pad (Fine Science Tools) and core body temperature was monitored using a rectal probe (Fine Science Tools), as described [27]. The head of the mouse was restrained in a stereotaxic frame, and the skull was exposed by making a mid-line incision in the scalp. The periosteum was removed and a circular incision was made in the parietal bone using a pneumatic dental drill to yield a cranial flap. The flap was lifted gently without damaging the dura mater underneath. Minor bleeding was controlled using gelfoam bits and the cranial window was bathed in pre-warmed artificial cerebrospinal fluid (aCSF) (132 mM NaCl, 2.95 mM KCl, 1.71 mM CaCl_2_.2H_2_0, 1.4 mM MgSO_4_, 6.7 mM Urea, 24.6 mM NaHCO_3_, 3.71 mM glucose, pH 7.4) [27]. The chamber was sealed with a coverslip held in place with vacuum grease.

### 2-photon intravital microscopy

All the intravital imaging experiments in this study were performed for up to 1.5 hours, as described previously [27]. Where extended recordings of more than 1.5 hours are required, we recommend the addition of a superfusion chamber containing aCSF as shown in Fig. S3 [27]. Imaging was performed using a LaVision Biotec Trimscope II single-beam 2-photon microscope (Bielefeld, Germany) attached to an Olympus BX-51 fixed-stage microscope equipped with 20× (NA0.8) water-immersion objective. The setup included external non-descanned dual-channel/fluorescence detectors and a diode-pumped, wide-band mode-locked Ti∶Sapphire femtosecond laser (MaiTai HP; Spectraphysics, 720–1050 nm, pulse length 140 fs; 90 MHz repetition rate). To label blood vessels, 800 µg of TRITC-conjugated dextran (Invitrogen) dissolved in saline or Evans blue conjugated to BSA was injected i.v. just prior to imaging. For data acquisition, firstly a suitable field of view was selected in the upper left region of the cranial window. The brain was exposed to polarized laser light at a wavelength of 900 nm, and x-y-t data of a 300 µm×300 µm plane at a resolution of 0.6 µm pixel^−1^ was captured at the rate of 1 frame per second. A minimum 120 to a maximum 600 frames were collected and sometimes combined with 3-dimensional z stacks to create x-y-z-t time-lapse images. The next field of view was recorded by moving horizontally across to the next 300 µm×300 µm plane within the cranial window. Only fields of view with at least 1 blood vessel were recorded. A minimum 29 to a maximum 123 blood vessels were assessed for each group of mice.

### Data analysis

Post-acquisition image analysis was carried out using Volocity software. Firstly, each blood vessel was annotated with a unique identity code. For the analysis of leukocyte behaviour, each blood vessel was initially assessed for intact blood flow present for the entire duration of the recording. Intact blood flow was defined by the presence of high velocity, freely flowing leukocytes (at least one free flowing cell during the observation period). The area of each blood vessel was derived by measuring its length and diameter. The phenotype of the blood vessels and its tributaries were assessed for brightness, size, central reflex, wall thickness and direction of flow [77]. As relying solely on phenotypic features can lead to misclassification of arteries and veins, we used a functional parameter that is widely utilised to distinguish the two. Diverging vessels with outflow of blood were classified as arteries and converging vessels with inflow of blood were classified as veins. Data were collated only from large post capillary venules. Leukocytes were tracked as they entered the field of view and over the entire observation period. Rolling cells were defined as single, round-shaped cells moving in the direction of the blood flow at a lower speed than free flowing cells. Adherent cells were defined as single cells that remain stationary for 30 seconds or longer. To normalise for variability in blood vessel diameter, the average number of rolling cells per mm^2^ of blood vessel over a period of 1 min and the number of adherent cells per mm^2^ of blood vessel were calculated. The mean rolling velocity (V_mean_) of leukocytes was defined as the distance travelled by rolling cells per second. The percentage of blood vessels within the cranial window with varying degrees of leukocyte infiltration, as defined in results below, was calculated.

### Statistical analysis

Differences in survival of treatment groups were analysed using the Mantel-Cox log-rank test. Correlations were calculated and plotted using Prism (Graphpad Prism software). For comparison of two groups, the Student's *t*-test (normally distributed) or the Mann-Whitney *U* test (not normally distributed) were used. For multiple comparisons, one-way ANOVA was used. A difference between groups was considered significant if p<0.05.

## Supporting Information

Figure S1 **Schematic depicts the progression of clinical disease during ECM.** The progression of clinical signs from ES to NS is shown.
(TIF)

Figure S2 **Relative percentages of BSL.** Uninfected and PbA-infected C57BL/6 mice with NS were sacrificed on day 7 p.i., and vascular perfusion was performed. (**A**) Gating strategy shows neutrophils (CD45^hi^, autofluorescence (AF)^lo^, Lin^−^, CD11b^+^, Ly6G^hi^) and inflammatory monocytes (CD45^hi^, AF^lo^, Lin^−^, CD11b^+^, Ly6G^−^, Ly6C^hi^) in the brains of uninfected and PbA-infected animals. (**B**) Gating strategy shows CD4^+^ T cells (CD45^hi^, AF^lo^, CD3^+^, CD4^+^) and CD8^+^ T cells (CD45^hi^, AF^lo^, CD3^+^, CD8^+^) as well as NK cells (CD45^hi^, AF^lo^, CD3^−^, CD335^+^, NK1.1^+^) (**C**) Monocytes, neutrophils, CD8^+^ T cells, CD4^+^ T cells and NK cells expressed as a percentage of the total CD45^hi^ brain leukocyte population pre- and post-PbA infection. Cells were harvested on day 7 p.i. when mice had ECM (n = 5 mice/group).
(TIF)

Figure S3 **Brain imaging model for ECM.** Picture depicts head restraint within the stereotaxic frame, cranial window preparation and a superfusion chamber that can be used for extended recordings of >1.5 hours.
(TIF)

Figure S4 **GFP^+^ leukocytes sequestering to the vascular endothelium are monocytes.** PbA-infected MacGreen mice with NS were sacrificed and brains were harvested. Whole mount brain sections were treated with anti-Ly6C or anti-Ly6G antibodies. Anti-CD31 antibodies were used for delineating the blood vessels. A series of single z-stack images were acquired by confocal microscopy. A representative single z-stack image shows the co-localisation of Ly6C but not Ly6G with GFP. Scale bars represent 10 µm.
(TIF)

Figure S5 **Locomotion pattern of monocytes during clinical progression of ECM.** (**A**) Quantitative analysis of the mean track velocity of monocytes during ES on day 5–6 p.i. (n = 5 mice) and (**B**) NS on day 7 p.i. (n = 3 mice). The speed at which monocytes travel per second in the blood vessel (instantaneous velocity) was calculated for (**C**) ES on day 5–6 p.i. and (**D**) NS on day 7 p.i. Three representative monocyte tracks are shown for each group. (**E**) Comparison of the V_mean_ of GFP^+^ monocytes in moderately and severely inflamed venules. Calculations were derived from 125 and 20 cell tracks. ****P<0.0001 (Mann-Whitney *U* test).
(TIF)

Figure S6 **Monocytes do not extravasate during ECM.** (**A**) Representative snapshot of monocytes rolling unidirectionally with blood flow (white arrows) during ES (n = 5 mice). Scale bar 30 µm. Path of monocytes are shown as purple tracks. (**B**) Individual cell tracking analysis plotted with each cell's start position at the origin and its movement along the x–y axis. (**C**) MI of GFP^+^ monocytes during ES (n = 5 mice) and NS (n = 3 mice). Calculations were derived from 126 and 23 cell tracks respectively. ns, not significant, Data are a mean of 3–5 independent experiments. (**D**) Groups of PbA-infected MacGreen×RAG^−/−^ recipient mice as in Fig. 4A were injected WGA-A594 i.v. just prior to sacrifice and then perfused intracardially. Brain sections were prepared. A series of single z-stack images of blood vessels with monocyte accumulation were acquired by confocal microscopy (n = 4 mice). **Panel (i)** The vascular lumen (outlined) is mostly devoid of F4/80^+^ cells (blue). **Panel (ii)** Monocytes marked bright red from WGA-A594 staining are F4/80^−^ or F4/80^lo^. **Panel (iii)** Circular GFP^+^ intravascular monocytes (yellow overlay) are F4/80^−^ or F4/80^lo^ (red arrowhead) whereas GFP^+^ perivascular cells (blue-green overlay) are mostly F4/80^+^ (yellow arrowhead). Scale bars 59 µm.
(TIF)

Figure S7 **Low monocyte accumulation in the brain microvasculature of PbA-infected MacGreen×RAG^−/−^ mice.** MacGreen×RAG^−/−^ mice were infected with PbA and intravital imaging was performed. (**A**) Representative snapshots of blood vessels. Scale bar 44 µm. (**B**) % blood vessels that have nil, moderate and severe levels of monocyte accumulation. (**C**) Average number of rolling and adherent monocytes per mm^2^ of endothelium min^−1^. Bars represent mean ± SEM. ****P<0.0001 (Mann Whitney *U* test), (n = 3–4 mice/group). Data are a mean of 2–3 independent experiments.
(TIF)

Figure S8 **Comparison of the V_mean_ of monocytes in inflamed venules.** Moderately and severely inflamed venules from MacGreen×RAG^−/−^ mice that received CD8^−^ splenocytes, naïve CD8^+^ T and primed CD8^+^ T cells were pooled and assessed. Calculations were derived from 97 and 127 cell tracks respectively. ****p<0.0001 (Mann-Whitney *U* test), (n = 7 mice/group). Data are a mean of 6 independent experiments.
(TIF)

Figure S9 **Dose-dependent effect of primed CD8^+^ T cells on clinical disease.** Primed CD8^+^ T cells (10, 4.5, 3 or 0×10^6^) were adoptively transferred into PbA-infected MacGreen×RAG^−/−^ recipient mice (n = 2–3 mice/group) and intravital imaging was performed on all groups on day 7 p.i. (**A**) survival and (**B**) % blood vessels with nil, moderate and severe levels of leukocyte accumulation are shown. A total 104, 62, 70 and 46 blood vessels were assessed for recipients of 10×10^6^, 4.5×10^6^, 3×10^6^ and 0×10^6^ primed CD8^+^ T cells respectively, with the following assessed per mouse: 10×10^6^ (n = 49, 33, 22), 4.5×10^6^ (n = 39, 23), 3×10^6^ (n = 37, 33) and 0×10^6^ (n = 10, 12, 24). Data are a mean of 2–3 independent experiments.
(TIF)

Figure S10 **Effect of monocyte depletion on the development of ECM – infection parameters.** Mice were infected with PbA and administered with CL or sham-treated (PBS) i.v. at the indicated times. (**A**) Haematocrit and (**B**) Parasitemia are shown.
(TIF)

Figure S11 **Genotyping profile of MacGreen×RAG^−/−^ mice.** (**A**) Pre-screening of peripheral blood leukocytes by flow cytometry shows the expression of GFP in the CD3^−^ fraction in both MacGreen×RAG^+/−^ and MacGreen×RAG^−/−^ mice. CD3^+^ T lymphocytes are seen in heterozygous but not in homozygous mice (**B**) Total DNA extracted from peripheral blood was analysed for RAG-1 expression by PCR. RAG^+/−^ mice express wildtype RAG as well as mutant RAG (Lane 2) whereas RAG^−/−^ mice express only mutant RAG (Lane 3).
(TIF)

Table S1 **Anaesthesia monitoring sheet.**
(DOCX)

Table S2 **List of antibodies.**
(DOCX)

Movi S1 **Nil leukocyte accumulation in the brain microvasculature.** A representative time-lapse sequence of an x–y plane shows the motility pattern of GFP^+^ leukocytes (green) in a non-inflamed blood vessel of uninfected MacGreen mice. Blood vessel is marked by infusion of TRITC-conjugated dextran (red). Perivascular myeloid cells line the blood vessels. GFP^+^ leukocytes flowed rapidly through the blood vessel appearing as ‘streaks’ and rarely interacted with the vascular endothelium. Leukocyte accumulation levels in such non-inflamed blood vessels (0–300 cells mm^2^ min^−1^) were graded as nil accumulation. This video as well as all others run at 1 frame/sec. Elapsed time is shown in hh:mm:ss.
(MOV)

Movie S2 **Moderate leukocyte accumulation in the brain microvasculature.** A representative time-lapse sequence of an x–y plane shows moderate levels of leukocyte accumulation in the brain microvasculature of PbA-infected MacGreen mice (300–1000 cells mm^2^ min^−1^).
(MOV)

Movie S3 **Severe leukocyte accumulation in the brain microvasculature.** A representative time-lapse sequence of an x–y plane shows severe levels of leukocyte accumulation in the brain microvasculature of PbA-infected MacGreen mice (>1000 cells mm^2^ min^−1^).
(MOV)

Movie S4 **GFP^+^ leukocytes sequestering to the brain microvasculature are not stained by isotype control antibody.** A time-lapse sequence of an x–y plane depicts GFP^+^ leukocytes in the brain microvasculature of PbA-infected MacGreen mice with NS. An Alexa647-conjugated isotype antibody was infused i.v. one-hour prior to start of recording. GFP^+^ leukocytes (green) did not take up any stain. White lines delineate blood vessels. Blue signals represent SHG from the collagen fibres in the brain parenchyma. Elapsed time is shown in hh:mm:ss.
(MOV)

Movie S5 **GFP^+^ leukocytes interacting with the brain vascular endothelium express Ly6C.** A representative time-lapse sequence of an x–y plane depicts GFP^+^ leukocytes in the brain microvasculature of PbA-infected MacGreen mice with NS. An Alexa647-conjugated Ly6C antibody was infused i.v. one-hour prior to start of recording. Stationary leukocytes have an orange punctate stain that overlay with GFP. Several rolling leukocytes also stained for Ly6C. White lines delineate blood vessels. Blue signals represent SHG from the collagen fibres in the brain parenchyma. Elapsed time is shown in hh:mm:ss.
(MOV)

Movie S6 **GFP^+^ leukocytes sequestering to the brain microvasculature do not express Ly6G.** A representative time-lapse sequence of an x–y plane depicts GFP^+^ leukocytes in the brain microvasculature of PbA-infected MacGreen mice with NS. An Alexa647-conjugated Ly6G antibody (red) was infused i.v. one-hour prior to start of recording. Leukocytes (green) did not take up any stain. White lines delineate blood vessels. Elapsed time is shown in hh:mm:ss.
(MOV)

Movie S7 **Rolling leukocytes in the dermal vasculature are not stained by isotype control antibody.** A representative time-lapse sequence of an x–y plane depicts GFP^+^ leukocytes (green) rolling in the dermal vessels of uninfected Lysozyme-GFP mice. An Alexa647-conjugated isotype antibody (red) was infused i.v. one-hour prior to start of recording. Leukocytes did not take up any stain. White lines delineate blood vessels. Elapsed time is shown in hh:mm:ss.
(MOV)

Movie S8 **Rolling leukocytes in the dermal vasculature express Ly6C.** A representative time-lapse sequence of an x–y plane depicts GFP^+^ leukocytes rolling in the dermal vessels of uninfected Lysozyme-GFP mice. An Alexa647-conjugated Ly6C antibody (red) was infused i.v. one-hour prior to start of recording. Leukocytes (green) show an orange punctate stain. White lines delineate blood vessels. Elapsed time is shown in hh:mm:ss.
(MOV)

Movie S9 **Rolling leukocytes in the dermal vasculature express Ly6G.** A representative time-lapse sequence of an x–y plane depicts GFP^+^ leukocytes (green) in the dermal vessels of uninfected Lysozyme-GFP mice. An Alexa647-conjugated Ly6G antibody (red) was infused i.v. one-hour prior to start of recording. A large number of leukocytes show a deep-red surface stain. White lines delineate blood vessels. Elapsed time is shown in hh:mm:ss.
(MOV)

Movie S10 **Rolling Monocytes do not exhibit extravasation.** A representative time-lapse sequence of an x–y plane shows rolling monocytes (green) in MacGreen mice 7 days p.i. Rolling monocytes were unidirectional and did not extravasate from the blood vessels. For visual clarity, blood vessels are not marked. Elapsed time is shown in hh:mm:ss.
(MOV)

Movie S11 **Monocyte accumulation levels in MacGreen×RAG^−/−^ mice following saline transfer.** A representative time-lapse sequence of an x–y plane shows the brain microvasculature in PbA-infected MacGreen×RAG^−/−^ mice 7 days post transfer of saline. Blood vessels are marked by infusion of TRITC-conjugated dextran. Monocytes flowed rapidly through the blood vessel appearing as ‘streaks’ and rarely interacted with the vascular endothelium. Saline transfer resulted in mostly nil accumulation (0–300 mm^2^ min^−1^). Elapsed time is shown in hh:mm:ss.
(MOV)

Movie S12 **Moderate monocyte accumulation levels in MacGreen×RAG^−/−^ mice.** A representative time-lapse sequence of an x–y plane shows moderate levels of monocyte accumulation in the brain microvasculature of PbA-infected MacGreen×RAG^−/−^ mice 7 days post-transfer of CD8^+^ T cells (300–1000 cells mm^2^ min^−1^).
(MOV)

Movie S13 **Severe monocyte accumulation levels in MacGreen×RAG^−/−^ mice.** A representative time-lapse sequence of an x–y plane shows severe levels of monocyte accumulation in the brain microvasculature of PbA-infected MacGreen×RAG^−/−^ mice 7 days post-transfer of CD8^+^ T cells (>1000 cells mm^2^ min^−1^).
(MOV)
