# Supplementary figures and images for: Real-Time Imaging Reveals the Dynamics of Leukocyte Behaviour during Experimental Cerebral Malaria Pathogenesis
Source: PLoS Pathog. 2014 Jul 17;10(7):e1004236. doi: 10.1371/journal.ppat.1004236 (PMC4102563; doi:10.1371/journal.ppat.1004236)

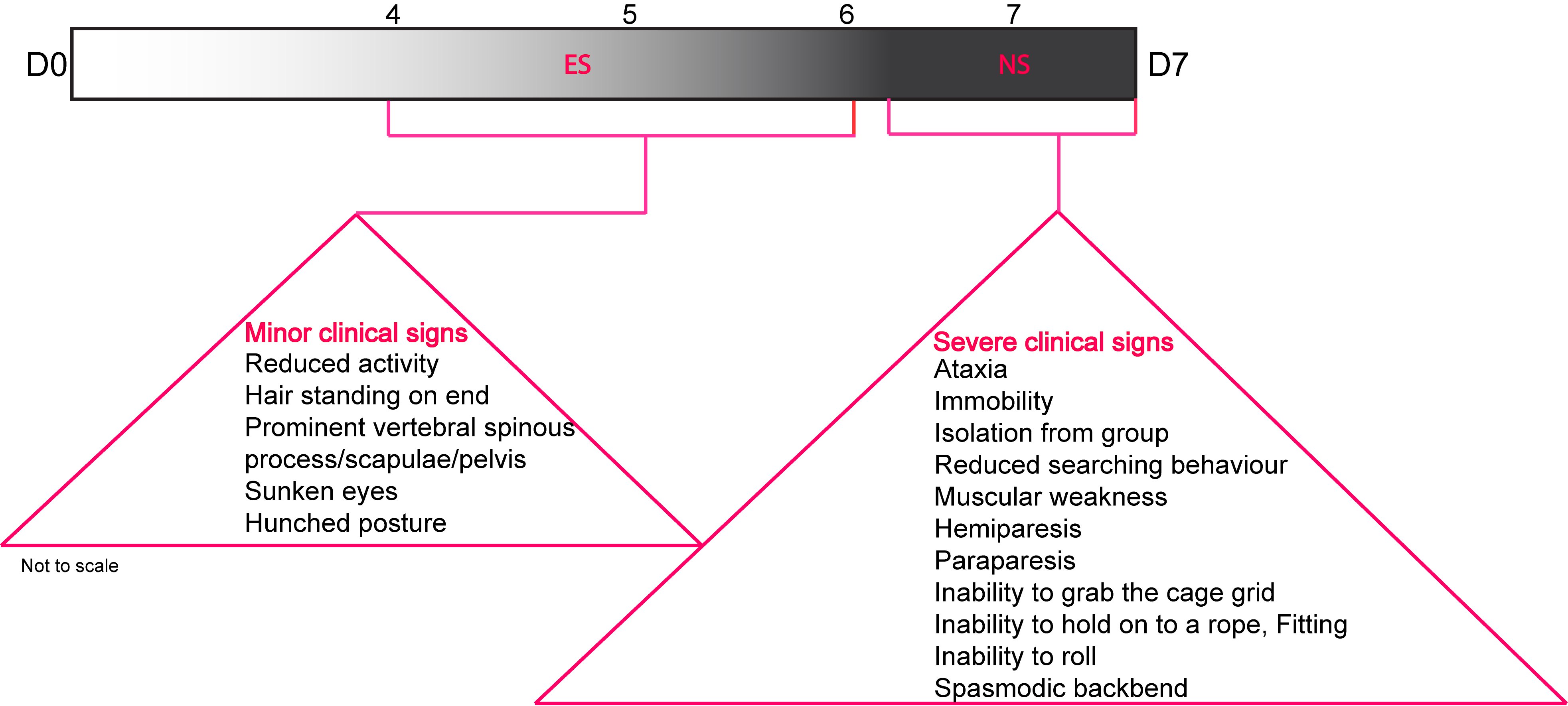

Supplement: Figure S1 — Schematic depicts the progression of clinical disease during ECM. The progression of clinical signs from ES to NS is shown. (TIF) [file ppat.1004236.s001.tif]

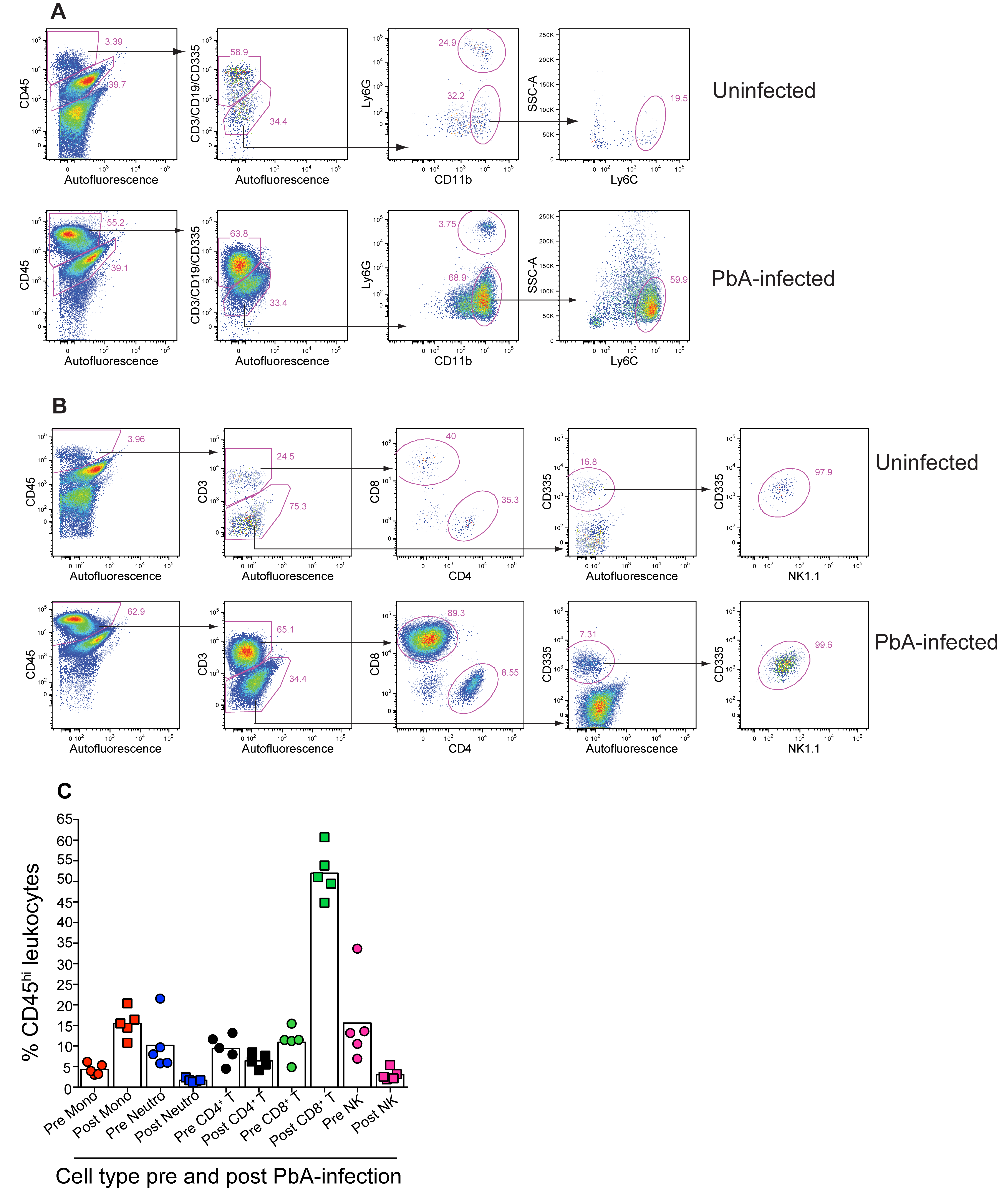

Supplement: Figure S2 — Relative percentages of BSL. Uninfected and PbA-infected C57BL/6 mice with NS were sacrificed on day 7 p.i., and vascular perfusion was performed. (A) Gating strategy shows neutrophils (CD45hi, autofluorescence (AF)lo, Lin−, CD11b+, Ly6Ghi) and inflammatory monocytes (CD45hi, AFlo, Lin−, CD11b+, Ly6G−, Ly6Chi) in the brains of uninfected and PbA-infected animals. (B) Gating strategy shows CD4+ T cells (CD45hi, AFlo, CD3+, CD4+) and CD8+ T cells (CD45hi, AFlo, CD3+, CD8+) as well as NK cells (CD45hi, AFlo, CD3−, CD335+, NK1.1+) (C) Monocytes, neutrophils, CD8+ T cells, CD4+ T cells and NK cells expressed as a percentage of the total CD45hi brain leukocyte population pre- and post-PbA infection. Cells were harvested on day 7 p.i. when mice had ECM (n = 5 mice/group). (TIF) [file ppat.1004236.s002.tif]

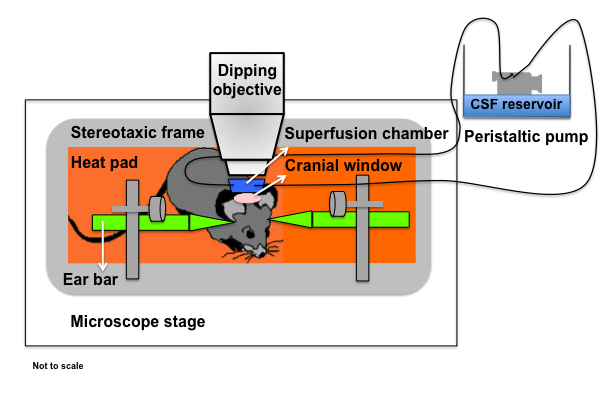

Supplement: Figure S3 — Brain imaging model for ECM. Picture depicts head restraint within the stereotaxic frame, cranial window preparation and a superfusion chamber that can be used for extended recordings of >1.5 hours. (TIF) [file ppat.1004236.s003.tif]

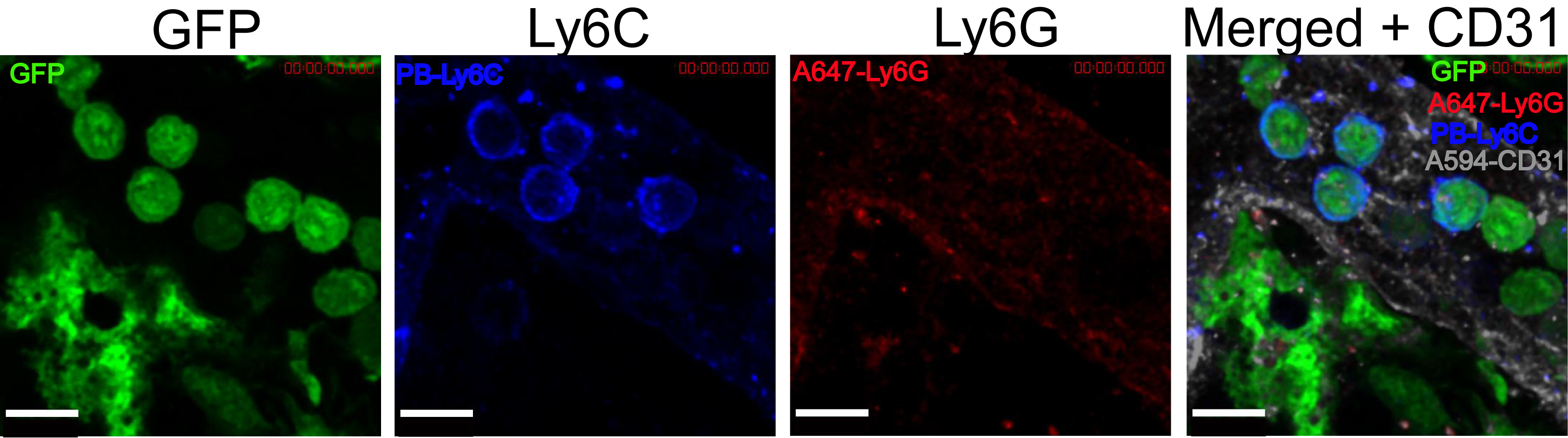

Supplement: Figure S4 — GFP+ leukocytes sequestering to the vascular endothelium are monocytes. PbA-infected MacGreen mice with NS were sacrificed and brains were harvested. Whole mount brain sections were treated with anti-Ly6C or anti-Ly6G antibodies. Anti-CD31 antibodies were used for delineating the blood vessels. A series of single z-stack images were acquired by confocal microscopy. A representative single z-stack image shows the co-localisation of Ly6C but not Ly6G with GFP. Scale bars represent 10 µm. (TIF) [file ppat.1004236.s004.tif]

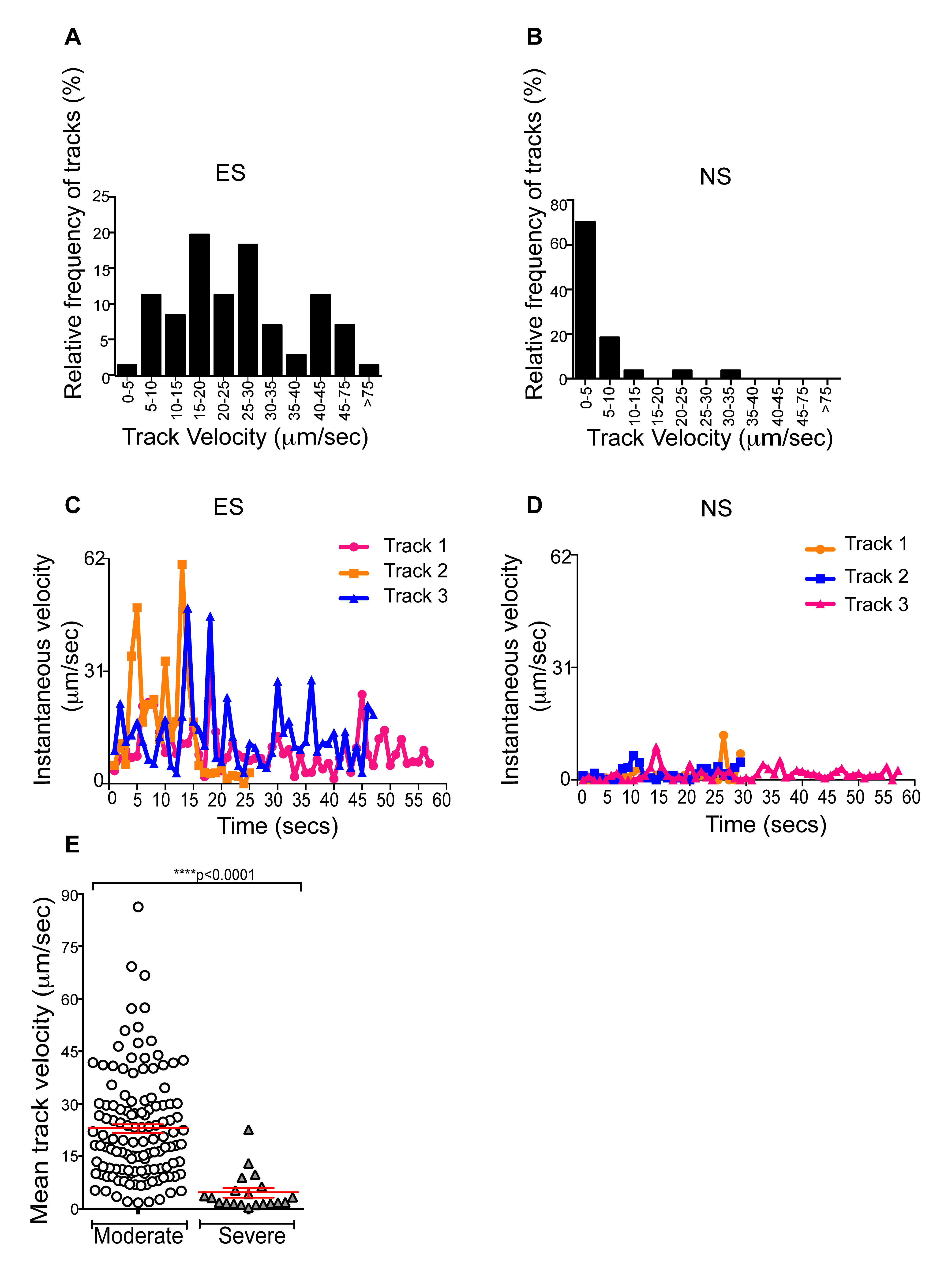

Supplement: Figure S5 — Locomotion pattern of monocytes during clinical progression of ECM. (A) Quantitative analysis of the mean track velocity of monocytes during ES on day 5–6 p.i. (n = 5 mice) and (B) NS on day 7 p.i. (n = 3 mice). The speed at which monocytes travel per second in the blood vessel (instantaneous velocity) was calculated for (C) ES on day 5–6 p.i. and (D) NS on day 7 p.i. Three representative monocyte tracks are shown for each group. (E) Comparison of the Vmean of GFP+ monocytes in moderately and severely inflamed venules. Calculations were derived from 125 and 20 cell tracks. ****P<0.0001 (Mann-Whitney U test). (TIF) [file ppat.1004236.s005.tif]

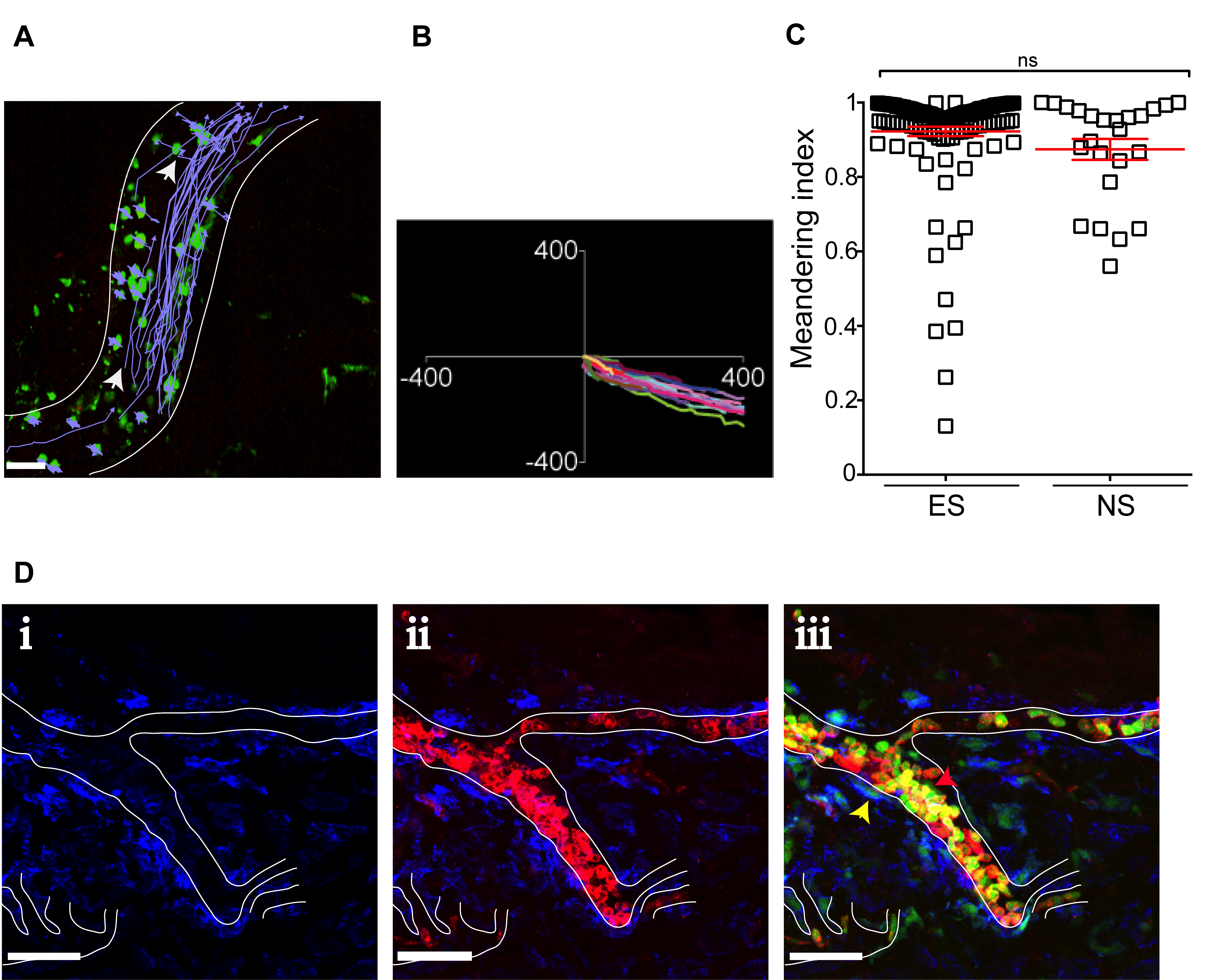

Supplement: Figure S6 — Monocytes do not extravasate during ECM. (A) Representative snapshot of monocytes rolling unidirectionally with blood flow (white arrows) during ES (n = 5 mice). Scale bar 30 µm. Path of monocytes are shown as purple tracks. (B) Individual cell tracking analysis plotted with each cell's start position at the origin and its movement along the x–y axis. (C) MI of GFP+ monocytes during ES (n = 5 mice) and NS (n = 3 mice). Calculations were derived from 126 and 23 cell tracks respectively. ns, not significant, Data are a mean of 3–5 independent experiments. (D) Groups of PbA-infected MacGreen×RAG−/− recipient mice as in Fig. 4A were injected WGA-A594 i.v. just prior to sacrifice and then perfused intracardially. Brain sections were prepared. A series of single z-stack images of blood vessels with monocyte accumulation were acquired by confocal microscopy (n = 4 mice). Panel (i) The vascular lumen (outlined) is mostly devoid of F4/80+ cells (blue). Panel (ii) Monocytes marked bright red from WGA-A594 staining are F4/80− or F4/80lo. Panel (iii) Circular GFP+ intravascular monocytes (yellow overlay) are F4/80− or F4/80lo (red arrowhead) whereas GFP+ perivascular cells (blue-green overlay) are mostly F4/80+ (yellow arrowhead). Scale bars 59 µm. (TIF) [file ppat.1004236.s006.tif]

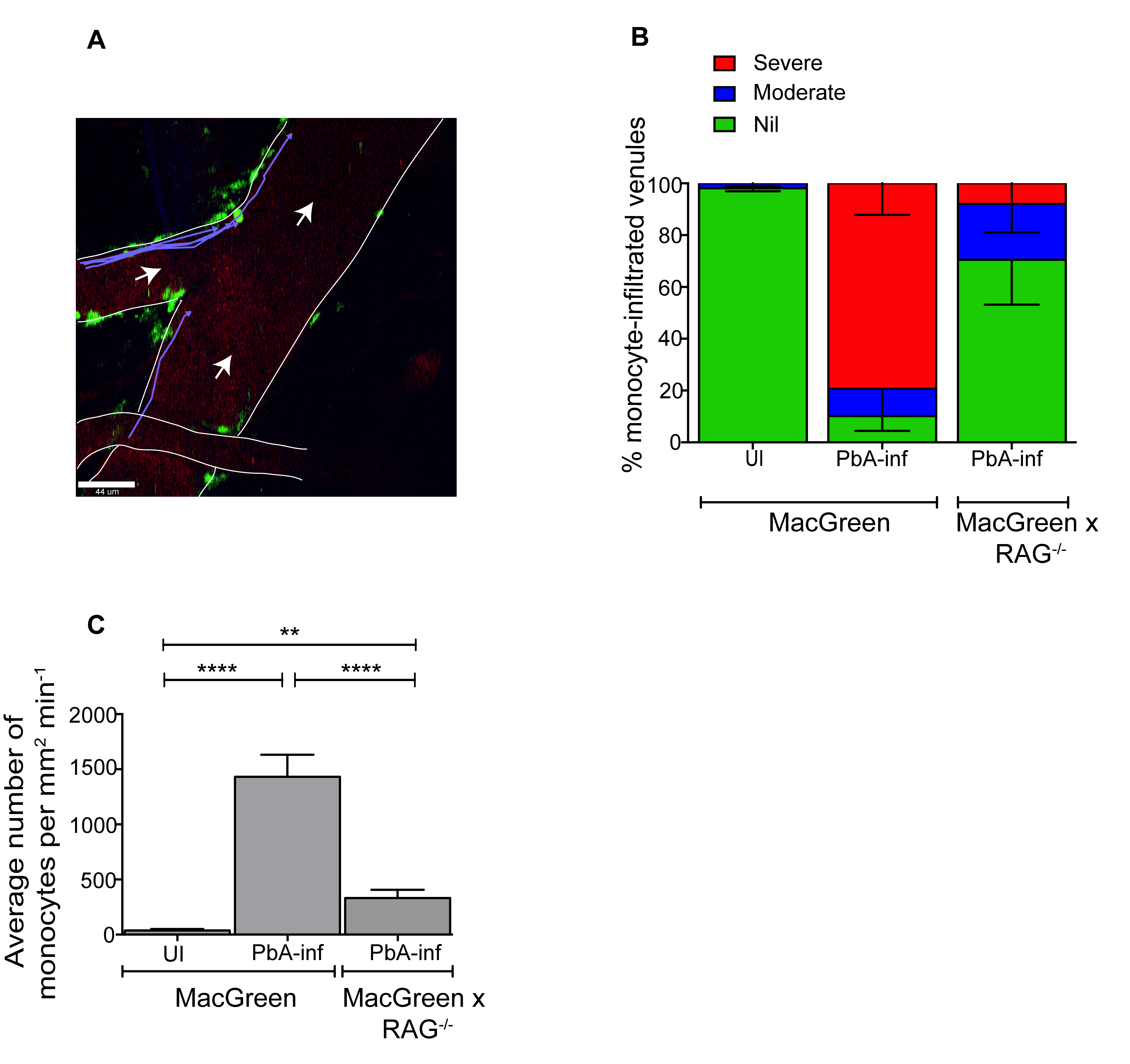

Supplement: Figure S7 — Low monocyte accumulation in the brain microvasculature of PbA-infected MacGreen×RAG−/− mice. MacGreen×RAG−/− mice were infected with PbA and intravital imaging was performed. (A) Representative snapshots of blood vessels. Scale bar 44 µm. (B) % blood vessels that have nil, moderate and severe levels of monocyte accumulation. (C) Average number of rolling and adherent monocytes per mm2 of endothelium min−1. Bars represent mean ± SEM. ****P<0.0001 (Mann Whitney U test), (n = 3–4 mice/group). Data are a mean of 2–3 independent experiments. (TIF) [file ppat.1004236.s007.tif]

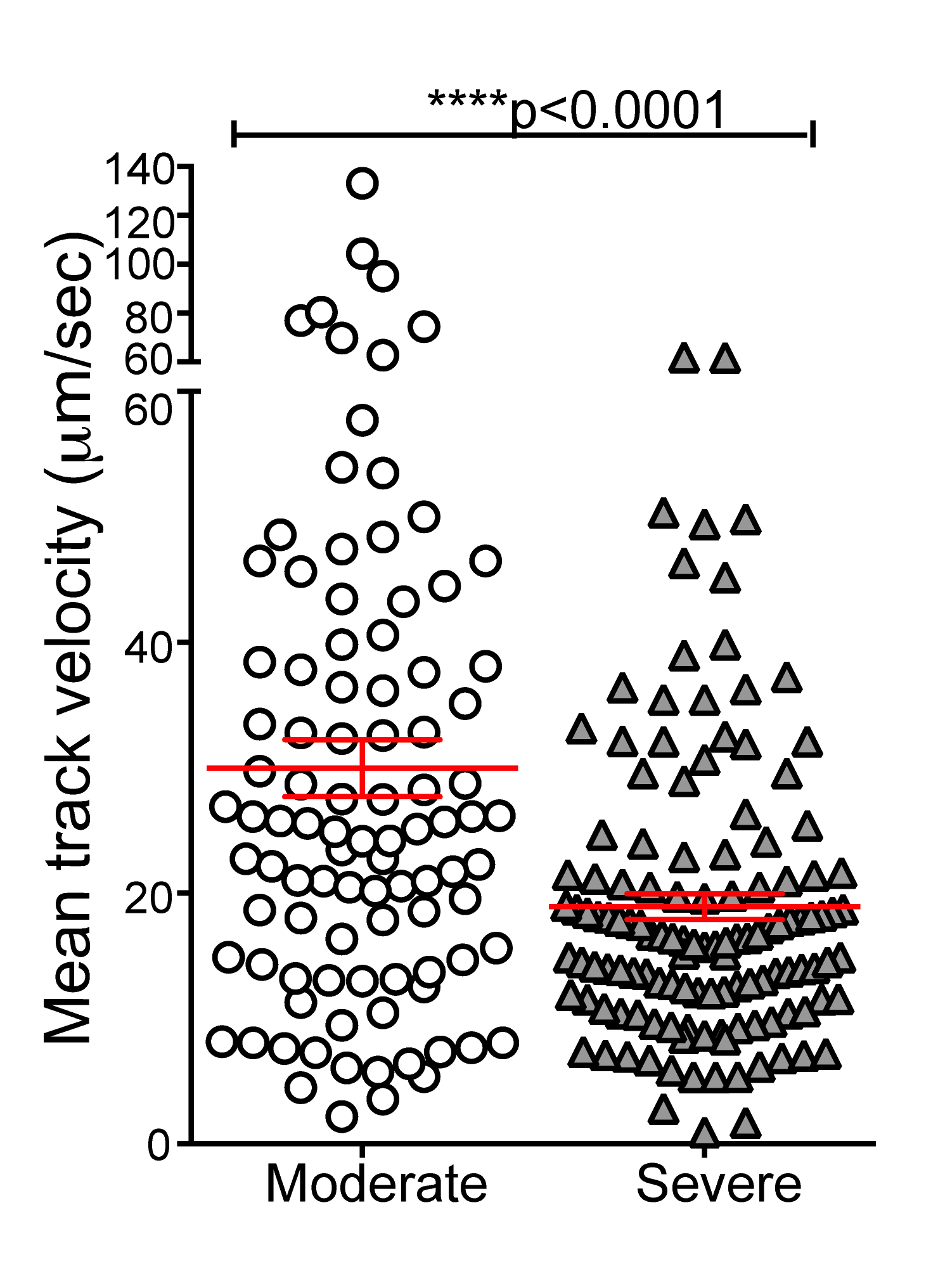

Supplement: Figure S8 — Comparison of the Vmean of monocytes in inflamed venules. Moderately and severely inflamed venules from MacGreen×RAG−/− mice that received CD8− splenocytes, naïve CD8+ T and primed CD8+ T cells were pooled and assessed. Calculations were derived from 97 and 127 cell tracks respectively. ****p<0.0001 (Mann-Whitney U test), (n = 7 mice/group). Data are a mean of 6 independent experiments. (TIF) [file ppat.1004236.s008.tif]

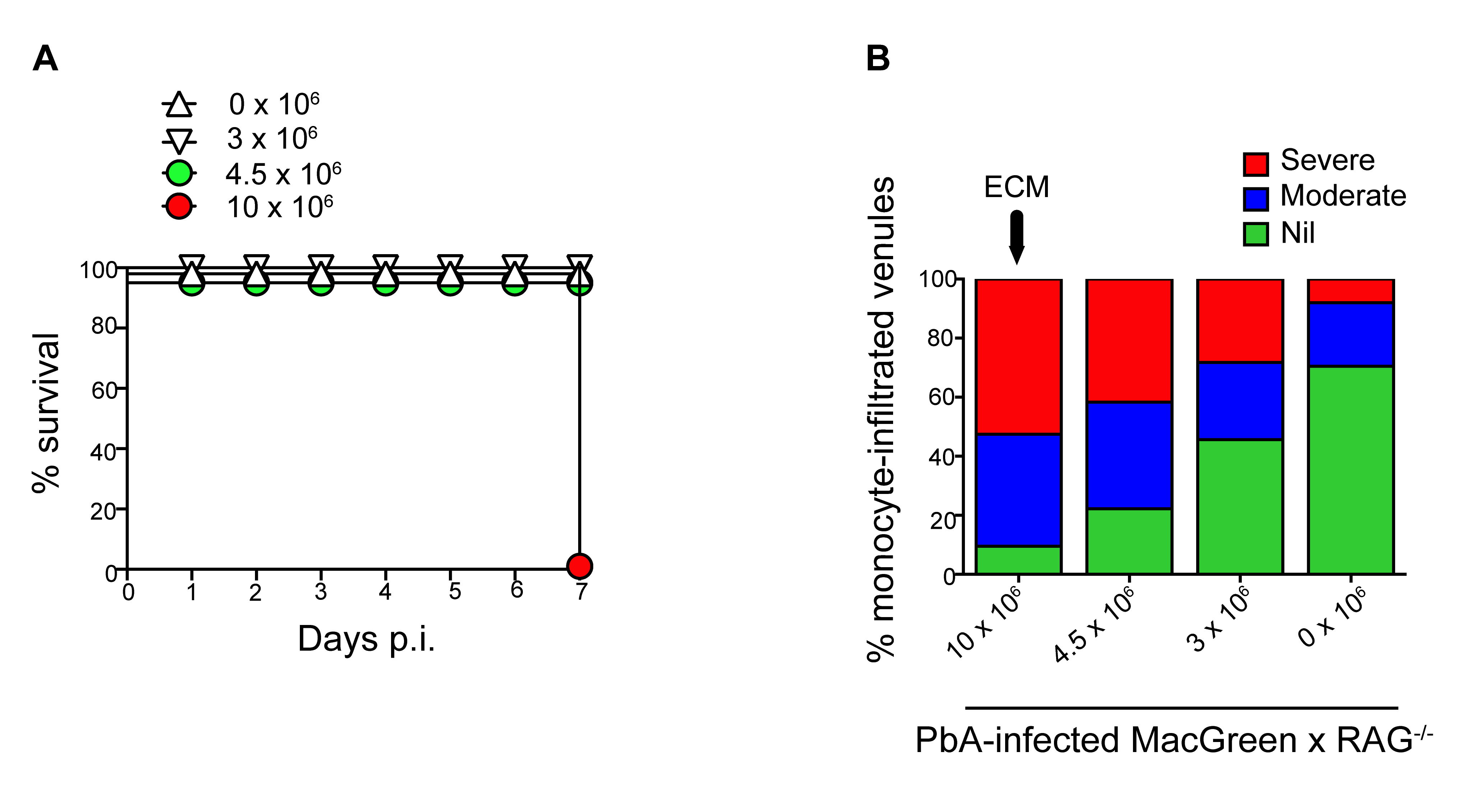

Supplement: Figure S9 — Dose-dependent effect of primed CD8+ T cells on clinical disease. Primed CD8+ T cells (10, 4.5, 3 or 0×10^6^) were adoptively transferred into PbA-infected MacGreen×RAG−/− recipient mice (n = 2–3 mice/group) and intravital imaging was performed on all groups on day 7 p.i. (A) survival and (B) % blood vessels with nil, moderate and severe levels of leukocyte accumulation are shown. A total 104, 62, 70 and 46 blood vessels were assessed for recipients of 10×106, 4.5×10^6^, 3×10^6^ and 0×10^6^ primed CD8+ T cells respectively, with the following assessed per mouse: 10×106 (n = 49, 33, 22), 4.5×10^6^ (n = 39, 23), 3×10^6^ (n = 37, 33) and 0×10^6^ (n = 10, 12, 24). Data are a mean of 2–3 independent experiments. (TIF) [file ppat.1004236.s009.tif]

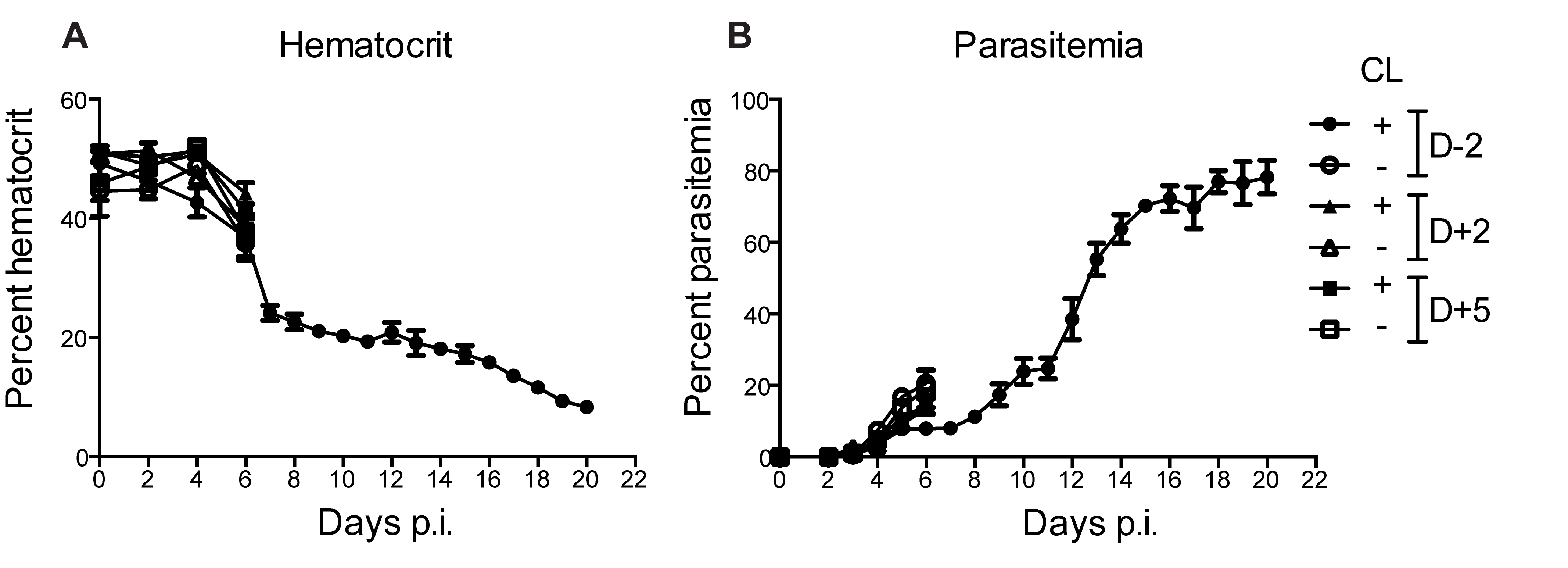

Supplement: Figure S10 — Effect of monocyte depletion on the development of ECM – infection parameters. Mice were infected with PbA and administered with CL or sham-treated (PBS) i.v. at the indicated times. (A) Haematocrit and (B) Parasitemia are shown. (TIF) [file ppat.1004236.s010.tif]

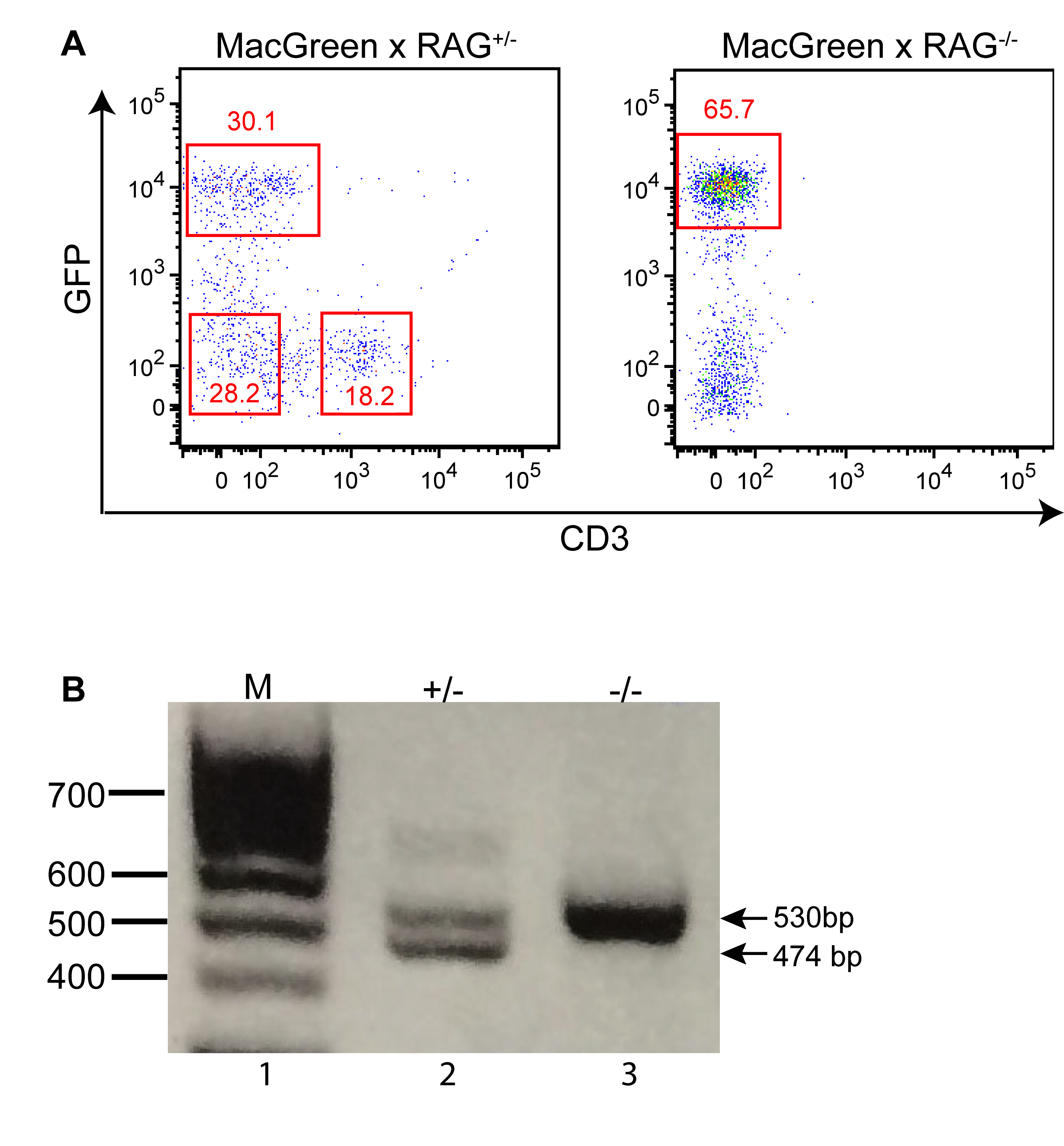

Supplement: Figure S11 — Genotyping profile of MacGreen×RAG−/− mice. (A) Pre-screening of peripheral blood leukocytes by flow cytometry shows the expression of GFP in the CD3− fraction in both MacGreen×RAG+/− and MacGreen×RAG−/− mice. CD3+ T lymphocytes are seen in heterozygous but not in homozygous mice (B) Total DNA extracted from peripheral blood was analysed for RAG-1 expression by PCR. RAG+/− mice express wildtype RAG as well as mutant RAG (Lane 2) whereas RAG−/− mice express only mutant RAG (Lane 3). (TIF) [file ppat.1004236.s011.tif]
