# Supplementary material for: Real-Time Imaging Reveals the Dynamics of Leukocyte Behaviour during Experimental Cerebral Malaria Pathogenesis
Source: PLoS Pathog. 2014 Jul 17;10(7):e1004236. doi: 10.1371/journal.ppat.1004236 (PMC4102563; doi:10.1371/journal.ppat.1004236)
Supplement: Table S1 — Anaesthesia monitoring sheet. (DOCX) [file ppat.1004236.s012.docx]

**TABLE S1: ANAESTHESIA MONITORING SHEET**

Protocol Number:____________________ EMERGENCY CONTACTS:

Animal ID: ________________________ Strain, age, sex: ________________________ Procedure undertaken: **INTRAVITAL BRAIN IMAGING OF MICE WITH ECM** Date __________

**Anaesthesia criterion to begin surgery: Animal still breathing, Normal respiration, loss of all reflexes, No signs of pain/**

| **Time of experiment** | **Animal still breathing** | **Respiration rate** | | | **Palpebral reflex** | **Whisker twitching reflex** | **Pedal-withdrawal reflex** | **Signs of pain observed** | **Signs of awareness observed** | **Any other signs of distress** |
| --- | --- | --- | --- | --- | --- | --- | --- | --- | --- | --- |
|  |  | **Low** | **Normal** | **Elevated** |  |  |  |  |  |  |
| **0 min** |  |  |  |  |  |  |  |  |  |  |
| **15 min** |  |  |  |  |  |  |  |  |  |  |
| **30 min** |  |  |  |  |  |  |  |  |  |  |
| **45 min** |  |  |  |  |  |  |  |  |  |  |
| **1 h** |  |  |  |  |  |  |  |  |  |  |

**awareness/distress**

**NOTES: Mice with ECM take 3-times as long as healthy mice to get anaesthetized**

Place a ✓ after testing breathing and respiration,

Place a ✗ after testing reflexes and signs.
