# Supplementary material for: Real-Time Imaging Reveals the Dynamics of Leukocyte Behaviour during Experimental Cerebral Malaria Pathogenesis
Source: PLoS Pathog. 2014 Jul 17;10(7):e1004236. doi: 10.1371/journal.ppat.1004236 (PMC4102563; doi:10.1371/journal.ppat.1004236)
Supplement: Table S2 — List of antibodies. (DOCX) [file ppat.1004236.s013.docx]

**TABLE S2: List of antibodies used for phenotyping**

| **Name of antibody** | **Conjugate** | **Clone** | **Supplier** |
| --- | --- | --- | --- |
| CD3 | BV421 | 145-2C11 | BD |
| CD3 | PerCPCy5.5 | 145-2C11 | BD |
| CD4 | APCCy7 | GK1.5 | BD |
| CD4 | PerCPCy5.5 | RM4-5 | BD |
| CD8 | APC | 53.6.7 | BD |
| CD8 | PE | 53-6.7 | BD |
| CD11b | APCCy7 | M1/70 | BD |
| CD19 | BV421 | 1D3 | BD |
| CD44 | APC | IM7 | BD |
| CD45 | PerCPCy5.5 | 30-F11 | BD |
| CD45 | APCCy7 | 30-F11 | BD |
| CD335 | V450/PE | 29A1.4 | BD |
| NK1.1 | FITC | PK136 | BD |
| B220 | NC650 | RA3-6B2 | eBioscience |
| Ly6G (for flow cytometry) | PE | 1A8 | BD |
| Ly6C (for flow cytometry) | FITC | HK1.5 | Biolegend |
| Ly6G (for *in vivo* labelling) | Alexa647 | 1A8 | Biolegend |
| Ly6C (for *in vivo* labelling) | Alexa647 | HK1.4 | Biolegend |
| F4/80  (for immunohistochemistry) | Biotin | BM8 | Jomar Bioscience |
| CD31  (for immunohistochemistry) | Purified | MEL13.3 | BD |
|  | Alexa594 |  | Life technologies |
| Ly6C  (for immunohistochemistry) | Biotin | HK1.4 | Biolegend |
|  | Streptavidin-Pacific blue |  | Life technologies |
| Ly6G  (for immunohistochemistry) | Alexa647 | 1A8 | Biolegend |
